# (Persistent) Organic pollutants in Germany: results from a pilot study within the 2015 moss survey

**DOI:** 10.1186/s12302-018-0172-y

**Published:** 2018-11-12

**Authors:** Annekatrin Dreyer, Stefan Nickel, Winfried Schröder

**Affiliations:** 1 Eurofins GfA GmbH, Air Monitoring, Stenzelring 14b, 21107 Hamburg, Germany; 2 University of Vechta, P.O.B. 1553, 49364 Vechta, Germany

**Keywords:** Biomonitoring, Atmospheric deposition, European moss survey, Halogenated flame retardants, HFR, BFR, Dioxins, Dechlorane Plus, DBDPE

## Abstract

**Background:**

Since 1990, every 5 years, moss sampling is conducted within the European moss monitoring programme to assess the atmospheric deposition of airborne pollutants. Besides many other countries, Germany takes regularly part at these evaluations. Within the European moss monitoring 2015, more than 400 moss samples across Germany were taken according to a harmonized methodology for the assessment heavy metal and nitrogen input. In a pilot programme, eight of these sites were chosen for additional investigations on a broad range of organic contaminants to evaluate their accumulation in moss and thereby their presence in atmospheric deposition in Germany. Target compound classes comprised polycyclic aromatic hydrocarbons (PAH), polychlorinated dibenzodioxins and –furans (PCDD/F), dioxin-like and non-dioxin-like polychlorinated biphenyls (dl-PCB, ndl-PCB), polyfluorinated alkyl substances, *classical* flame retardants as well as *emerging* chlorinated and brominated flame retardants. In total, 120 target compounds were analysed. For some analytes, comparisons of accumulation in moss and tree leave samples were possible.

**Results:**

Except for certain flame retardants, PFAS, and ndl-PCB, substances of all other compound classes could be quantified in moss samples of all sites. Concentrations were highest for PAH (40–268 ng g^−1^) followed by *emerging* flame retardants (0.5–7.7 ng g^−1^), polybrominated diphenyl ethers (PBDE; 0.3–3.7 ng g^−1^), hexabromocyclododecane (HBCD; 0.3–1.2 ng g^−1^), dl-PCB (0.04–0.4 ng g^−1^) and PCDD/F (0.008–0.06 ng g^−1^).

**Conclusions:**

Results show the widespread atmospheric distribution and deposition of organic contaminants across Germany as well as the suitability of moss as bioaccumulation monitor for most of these compound classes. Compared to nearby tree leaf samples, accumulation potential of moss appeared to be higher for pollutants of high octanol–air partition coefficient (K_OA_) and octanol–water partition coefficient (K_OW_).

**Electronic supplementary material:**

The online version of this article (10.1186/s12302-018-0172-y) contains supplementary material, which is available to authorized users.

## Background

Monitoring and mapping of atmospheric deposition can be achieved by chemical transport models, technical sampling devices and bioaccumulators such as moss [38]. Due to a lack of roots, mosses receive pollutants rather from the air than from substrates. Pollutant absorbtion through the leaf surface of mosses is facilitated as a waxy cuticle is missing [60]. Their accumulation is further aided by the high surface to volume ratio of moss [24, 61]. Furthermore, mosses grow in various habitats around the world, even in Polar Regions. Therefore, mosses were and are applied as biomonitors, mainly for nitrogen and heavy metals, but also, although less often, for organic contaminants [7, 19, 20, 24, 31, 43, 44, 46, 50, 52]. Up to now, only few studies were published regarding organic pollutants in moss specimens from Germany [50, 52].

Among organic contaminants, persistent organic pollutants (POP) are compounds which were already identified as substances of environmental as well as human concern. Besides their persistence, POP are toxic to humans and wildlife, accumulate significantly in living organisms and are transported over long distances [49]. After emissions to the environment, POP become globally distributed, also to regions where they have not been used.

Several chemicals are listed as POP by the Stockholm Convention. These compounds usually share a high degree of halogenation. Among them are some organochlorine pesticides, polychlorinated biphenyls (PCB), polychlorinated dibenzodioxins and –furans (PCDD/F), polybrominated diphenyl ethers (PBDE), hexabromobiphenyl (HBB), hexabromocyclododecane (HBCD) or perfluorooctane sulfonic acid (PFOS). Some POP are unintentionally formed, e.g. PCDD/F by combustion of chlorine-containing substrate or as by-products of industrial processes. Others were intentionally produced for applications as pesticides or industrial chemicals, e.g., PCB as heat exchange fluid or additive in paints and plastics, PFOS as coating additive, or HBCD, HBB, and PBDE as flame retardants [49]. Production or applications of POP are regulated or restricted; however, market demands often require adequate replacements. For example, bis(2-ethylhexyl)-tetrabromophthalate (BEHTeBP) and 2-Ethylhexyl-2,3,4,5-tetrabromobenzoate (EHTeBB) were used to replace PentaBDE. Decabromodiphenyl ethane (DBDPE) or Dechlorane Plus (DP) were introduced as alternative for DecaBDE [47, 58]. Although such substitutes might not meet all POP criteria, they often share properties as those substances they were designed to replace and thus might be similarly harmful to the environment or to humans. Therefore, it is not only important to monitor classical POP but also to investigate their substitutes or compounds with (anticipated) POP-like properties.

This study aims for reporting on the determination of selected organic contaminants in moss samples from Germany taken within the framework of the 2015 European moss survey. In the European moss survey programme, since 1990, every 5 years, moss have been sampled at up to about 7300 sites in up to 36 countries, among them Germany. Within the European moss survey programme, sampling, chemical determination of heavy metals (since 1990), nitrogen (since 2005), as well as POP and POP-like substances (since 2010) in moss specimens, quality control and statistical evaluation were conducted according to a harmonized methodology [25]. In Germany, (persistent) organic pollutants in moss samples were to be determined for the first time within this programme in 2015. Therefore, this study aims for the determination of a wide range of organic pollutants to get insight of their extent of accumulation and distribution in moss from Germany. As some of these contaminants were also monitored in tree leaf samples within the German Environmental Specimen Bank (ESB), accumulation in moss should be compared to that in tree leaves. Among the target analytes of this study are polycyclic aromatic hydrocarbons (PAH) including benzo(a)pyrene (BaP) as classical air pollutant with limit value of 1 ng m^−3^ in ambient air (PM10) [15], PCDD/F, PCB, perfluoroalkyl substances (PFAS), PBDE as well as *novel* or *emerging* halogenated flame retardants (HFR) of different substance classes (Dechloranes, brominated aromates, brominated ethers, cyclic HFR). Overall, 120 target analytes were investigated.

## Methods

### Sampling

**Fig. 1:**
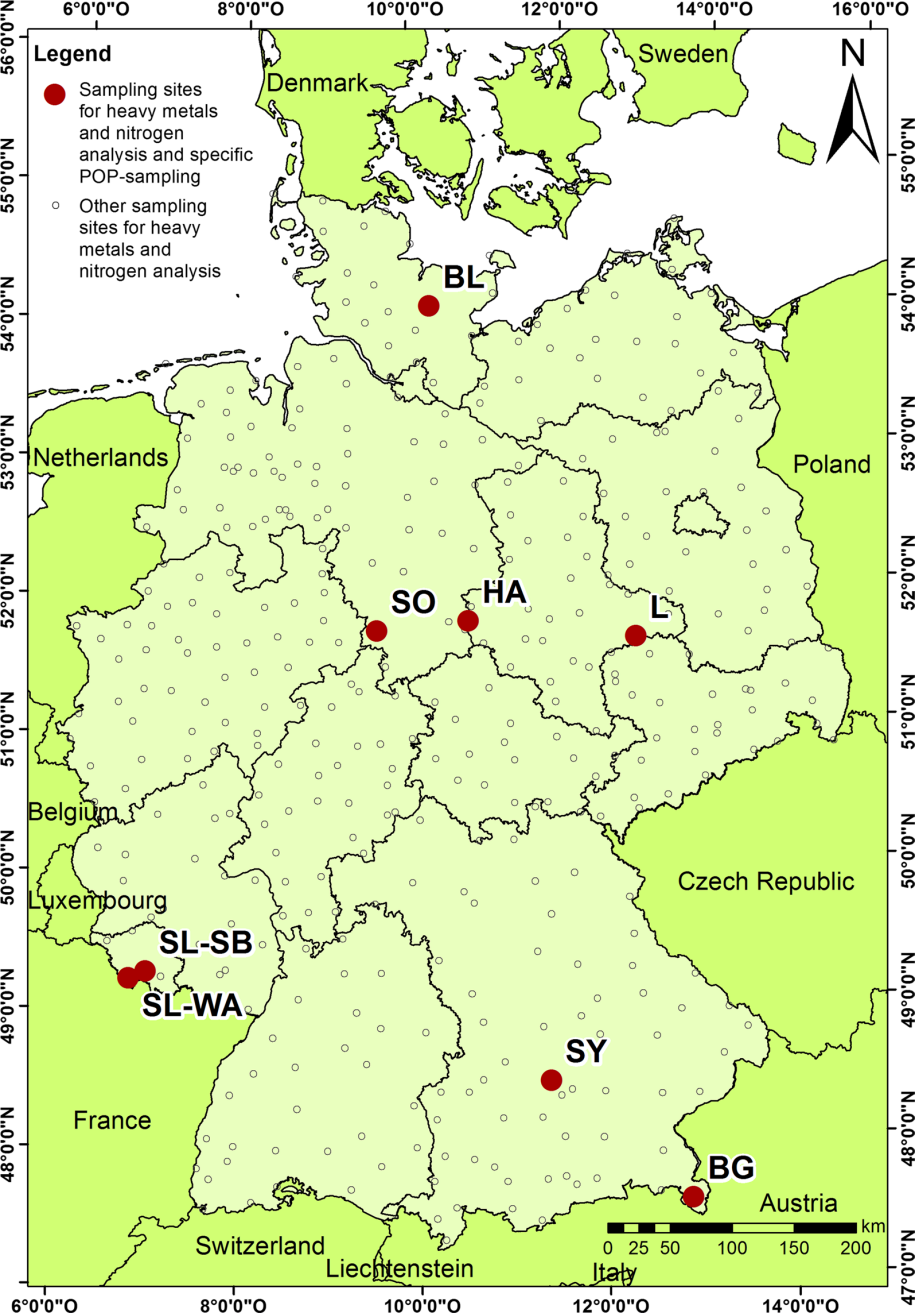
Location of the moss sampling sites investigated for organic pollutants within the 2015 moss survey. *BL* Bornhöved Lakes region, *SO* solling, *HA* Harz, *L* Leipzig conurbation, *SL-SB* Saarland Conurbation close to Saarbrücken, *SL-WA* Saarland conurbation close to Warndt recreation area, *SY* Bavarian tertiary uplands close to Scheyern, *BG* Berchtesgaden

The moss survey manual [56] recommends the sampling of moss near long-term atmospheric monitoring stations. Within the statistically designed moss monitoring network covering Germany [39], moss samples for the analysis of organic contaminants were taken in November 2016 at eight sites which were located close to areas where tree leaves as bioindicators of atmospheric pollution are routinely sampled within the ESB programme of the German Environment Agency. Tree sampling sites of the German ESB programme represent four different types of terrestrial ecosystems (near-natural, forestry, agricultural, conurbations). Moss sampling sites were chosen so that each of these ecosystem types were represented by at least one moss sampling site. Sites were located at Berchtesgaden/Wimbachtal (BG; Germany’s only high mountain national park in the Alps), Bavarian tertiary uplands/Scheyern (SY; part of the southern German basin), Halle/Leipzig-conurbation/Dübener Heide (HL; the chemical triangle of central Germany, formerly eastern Germany), Bornhöved Lakes region/Belauer See (BL; part of the northern German basin and the main water divide between North- and Baltic Sea), Solling/Sievershausen (SO; second highest and largest low mountain range), Harz/Ilsenburg (HA; Germany’s largest forest national park) and Saarland (an old-industrialized conurbation) at a forest preserve close to Saarbrücken (SL-SB) and at Waldgassen close to the Warndt recreation area (SL-WA) (Fig. 1, Additional file 1: Table S1). Target moss species were *Pleurozium schreberi*, *Hypnum cupressiforme* or *Pseudoscleropodium purum* (Additional file 1: Table S1). The selection of these moss species as to be sampled by priority is discussed by Fernandez et al. [17], Harmens et al. [26], Holy et al. [28], and Schröder et al. [48]. Moss samples were taken at sites representative of non-urban areas in general accordance to the ICP vegetation moss monitoring manual [25, 56]. Sampling occurred in a distance of at least 300 m from main roads, villages and industries and at least 100 m away from smaller roads and houses. Sites were situated at least 3 m away from the nearest tree canopy. Samples were taken using pre-cleaned 2.5 L wide mouth amber glass bottles. At each site, two of these bottles were filled with moss. Samples were transported and stored between 0 and 4 °C and in dark until preparation. Storing temperature during transport was monitored.

### Sample preparation

Within 2 weeks after sampling, moss specimens were cleaned off adhering material such as grass, needles, leaves, or soil. Obvious non-target-moss species were also removed. Samples were not washed. Green and green-yellowish moss shoots were separated for analysis using pre-cleaned stainless steel tweezers. Samples were stored at − 20 °C until drying, homogenisation and further analysis.

### Analyses

For the analysis of the 120 target compounds, several individual analytical procedures were used. The methods are routinely applied analytical procedures according to international guidelines and/or are published elsewhere. Therefore, they are only briefly described here. All target analytes and their abbreviations are listed in the Additional file 1.

#### PAH

3 g of dried moss were spiked with mass-labelled PAH and extracted using acetone and Soxhlet extraction. After extraction, samples were shaken out with *n,n*-dimethylformic amide and cyclohexane followed by a column chromatography clean-up using silica. The 16 EPA-PAHs were determined by high-resolution gas chromatography coupled to high-resolution mass spectrometry (HRGC–HRMS) and quantified using the isotope dilution method [22].

#### PCDD/F and PCB

Samples were analysed according to a method described by Neugebauer et al. [37]. 2 g of dried moss were spiked with mass-labelled PCDD/F and extracted using toluene and Soxhlet extraction with at least 60 extraction cycles. Extracts were cleaned-up by multi-layer column chromatography using acidic silica and aluminium oxide. Samples were measured by HRGC–HRMS and quantified using the isotope dilution method. Seventeen 2,3,7,8-substituted PCDD/F congeners, 12 dioxin-like PCB (dl-PCB) and six non dioxin-like PCB (ndl-PCB; PCB 28, 52, 101, 138, 153 und 180) as well as toxicity equivalents were determined.

#### PFAS

About 0.5 g of dried moss were spiked with mass-labelled PFAS and extracted using methanol and ultra-sonication. Samples were cleaned up using Envicarb. 16 PFAS (PFBS, PFHxS, PFHpS, PFOS, PFDS, PFBA, PFPeA, PFHxA, PFHpA, PFOA, PFNA, PFDA, PFUnA, PFDoA, PFTrA, PFTeA) were determined by high-performance liquid chromatography using electrospray ionisation tandem mass spectrometry (HPLC–ESI–MS/MS) and quantified using the isotope dilution method [18].

#### HBCD and polybrominated biphenyls (PBB)

On the basis of a method by Fliedner et al. [18], about 5 g of dried moss were spiked with mass-labelled HBCD and extracted using toluene and Soxhlet extraction for at least 12 h. After extraction, concentrated sulfuric acid was added for clean-up. Samples were further cleaned by column chromatography using aluminium oxide. The three HBCD isomers (α, β, γ-HBCD) were determined by HPLC–ESI–MS/MS. Seven PBB (PBB 52, 101, 153, 180, 194, 206 und 209) were determined by gas chromatography coupled to mass spectrometry (GC–MS). All samples were quantified using the isotope dilution method.

PBDE and HFR: Samples were analysed according to a method described by Neugebauer et al. [36]. Briefly, nineteen mass-labelled standards were added to each sample of about 4 g dried moss prior to accelerated solvent extraction with hexane:dichloromethane 1:1 (v:v). Samples were cleaned-up by a multicolumn procedure involving Na_2_SO_4_ upon 2 g silica, BioBeads SX-3, and Florisil (5% deactivated). Instrumental detection of 19 HFR (TBA, ATE, BATE, DPTE, BTBPE, EHTeBB, BEHTBP, PBT, HBBz, PBEB, DBDPE, Dec602, Dec603, Dec604, syn-DP, anti-DP, Cl_10_anti-DP, Cl_11_anti-DP, DPMA) occurred by gas chromatography coupled to tandem mass spectrometry using atmospheric pressure ionization (GC-API-MS/MS). GC–MS was applied to determine 24 PBDE (BDE 17, 28, 47, 49, 66, 71, 77, 85, 99, 100, 119, 126, 138, 153, 154, 156, 183, 184, 191, 196, 197, 206, 207, 209) in a separate run. For the BL site, PBDE and HFR could not be determined as the amount of moss was not high enough to enable this additional analysis.

### QA/QC

Sampling equipment was machine-washed, thoroughly rinsed using methanol, acetone, and hexane and heated to 250 °C for 12 h. The glassware used for laboratory analysis was machine-washed, heated at 300 °C, and washed with acetone before use. To shield the partially UV-sensitive flame retardants from UV radiation, light exclusion was performed (e.g., by alumina foil, closing shutters, keeping off unnecessary halogen lights, using brown glassware as far as possible). Overall, 78 mass-labelled internal standards were used to correct for losses and irregularities during analysis and measurement. Recovery rates of mass-labelled standards in individual samples were generally in the acceptable range as required by EPA 1614A [57] (25–150%, BDE 209 up to 200%) with few exceptions for the most volatile (e.g. 10% NapD_8_) or highly halogenated target analytes (e.g. 250% ^13^C DBDPE). Sample analyses were performed at laboratories which were accredited according to DIN EN ISO IEC 17025 [9] and regularly take part in round robin tests. Except for the PBDE/HFR analysis, all analytical procedures were accredited. The method for the analysis of PBDE and HFR was extensively validated [36]. Certified reference material (EDF2525, T665QC) or similarly materials of known target analyte concentrations as well as blank samples were analysed to check the analytical performance. Method quantification limits (MQL) were calculated on the basis of overall laboratory blank concentrations and signal intensity and varied depending on the used sample amounts. With few exceptions, concentrations in blank samples analysed within the moss sample sequences were below these MQL. Exceptions (selected ndl-PCB and PFAS or PAH) did not impact the findings as the chromatograms of these blank samples did not show any significant influence on the analytical parameters in the range of the sample signals. Instruments used for detection and quantification were regularly checked in terms of separation performance, resolution and sensitivity. Calibrations were performed as multi-point calibrations.

## Results and discussion

### Potential constraints

Even if the moss technique is widely accepted as reliable tool for monitoring and mapping of atmospheric deposition across areas of large spatial extent, there are critical comments, e.g., those compiled by Fernandez et al. [17]. The use of different moss species was discussed as important factor influencing the concentrations of elements in moss and contributing to the variability observed. Additionally, the statistical relevance of sampling site characteristics (moss species, canopy drip effect, etc.) and of their surroundings (land use, atmospheric deposition, etc.) and boundary conditions of data production (sampling, chemical/physical methods, etc.) are important and were evaluated, e.g. for Germany and Europe by Harmens et al. [25], Holy et al. [28], Nickel et al. [40], or Schröder et al. [48]. If and to which significance these factors may also affect organic contaminants accumulated by moss is yet unclear and may be subject of future investigations.

### Concentrations of organic pollutants in moss

**Fig. 2:**
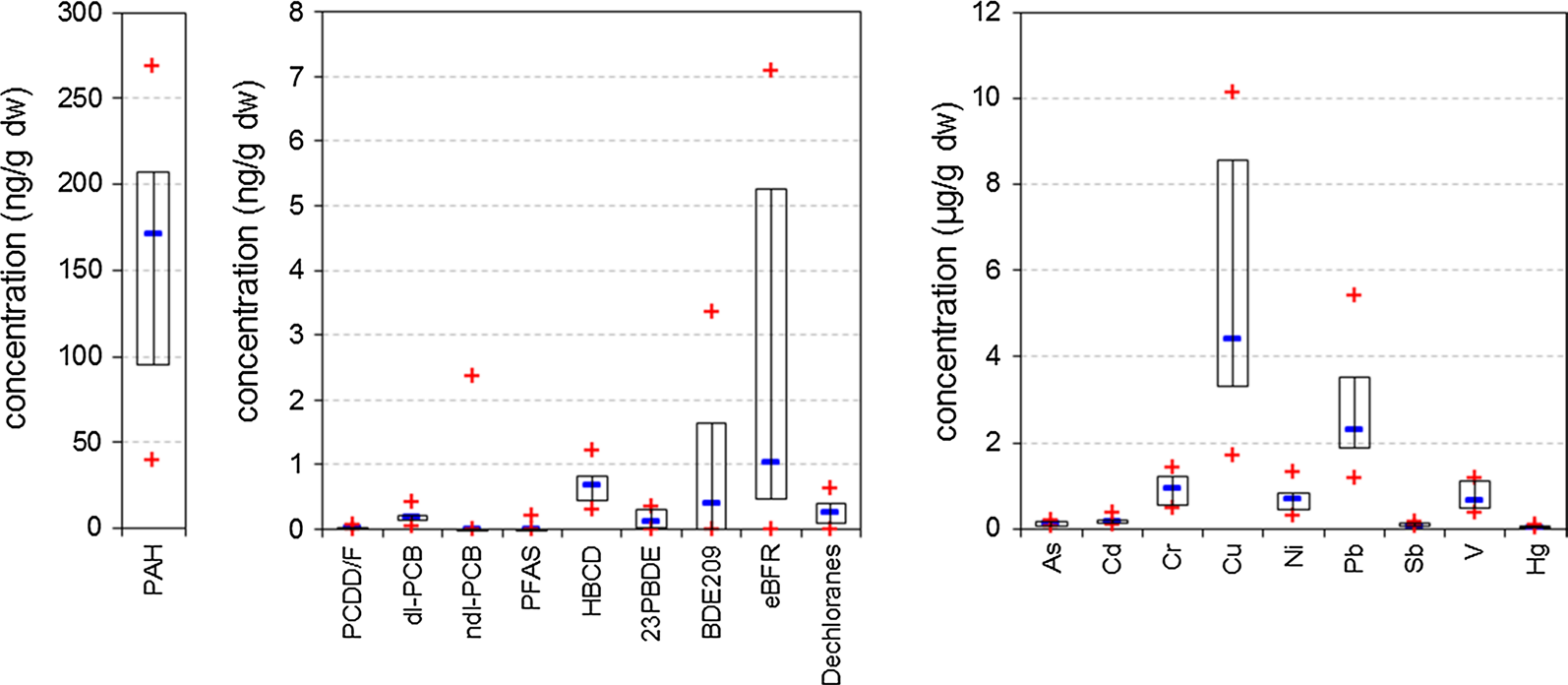
Overview about total concentrations of groups of organic contaminants in investigated moss samples in comparison to heavy metal concentrations. Note the different scales and units. +: minimum/maximum concentration, −: median concentration, box: 25%/75% percentile

Figure 2 depicts an overview about the total concentrations of several groups of organic contaminants in comparison to those of heavy metals. Concentrations of heavy metals were at least one order of magnitude higher than those of the organic compounds. Among the organic pollutants, PAH were observed at highest concentrations followed by flame retardants. In contrast to these substance groups, concentrations of PCDD/F, PCB, and PFAS were much lower or below the MQL. Median concentrations of flame retardants decreased in the order of eBFR, HBCD, BDE209, Dechloranes, and remaining PBDE. In the following, substance classes of the investigated pollutants will be discussed separately.

#### PAH

**Fig. 3:**
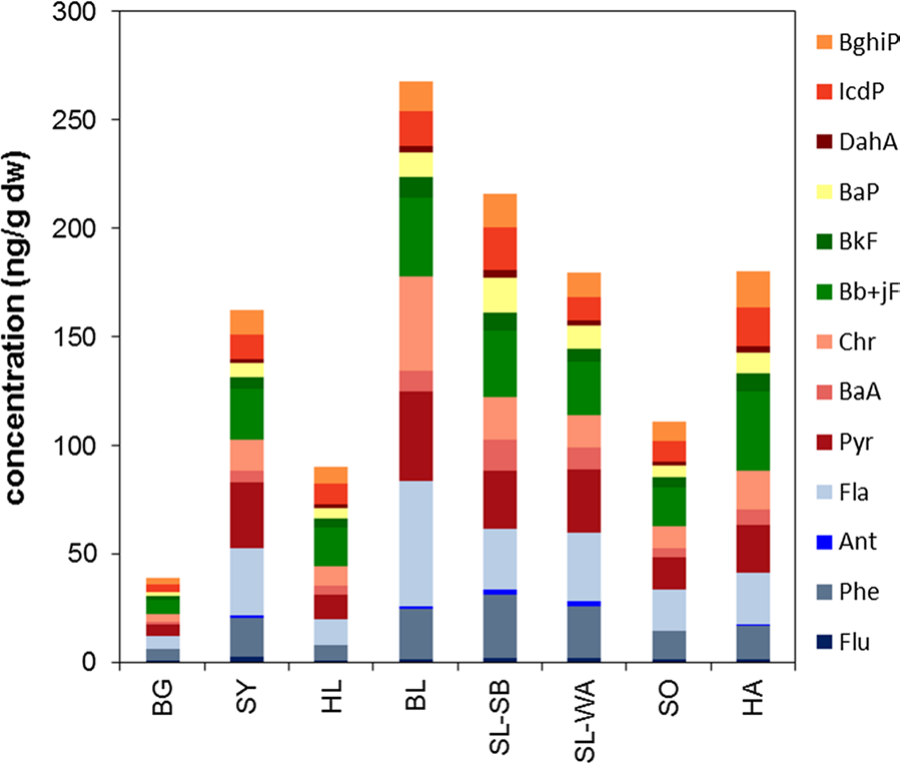
PAH concentrations (ng g^−1^ dw) observed in German moss samples

PAH are the most frequently investigated organic contaminants in moss samples. PAH concentrations in investigated moss samples of the present study are given in Fig. 3, Additional file 1: Figure S1 and Tables S5, S6. Total concentrations were between 39.3 ng g^−1^ dry weight (dw) (4.2 ng g^−1^ fresh weight (fw)) at BG and 267.8 ng g^−1^ dw (47.7 ng g^−1^ fw) at BL. BaP as marker PAH was observed at concentrations between 1.7 ng g^−1^ dw (0.2 ng g^−1^ fw; BG) and 16 ng g^−1^ dw (SL-SB) or 2 ng g^−1^ fw (BL). German moss concentrations were mostly higher than those observed 2015 in moss samples (*Pleurozium schreberi* or *Hylocomium splendens*) in Sweden (15–120 ng g^−1^ dw) [8]. Total PAH as well as BaP concentrations observed in German moss samples were in the same order as concentrations for PAH (Σ13PAH; 149–360 ng g^−1^ dw, 100–356 ng g^−1^ dw, 98–698 ng g^−1^ dw, respectively) and BaP (6–17 ng g^−1^ dw, 2–6 ng g^−1^ dw, 3–47 ng g^−1^ dw, respectively) observed in *Hypnum cupressiforme* sampled in 2010 in France, Spain, and Switzerland [20]. They were similarly high compared to concentrations for Σ16 PAH observed in *Hypnum cupressiforme* moss bag samples from an agrarian area in Italy (Σ16 PAH 60–95 ng g^−1^ dw) [6] or in moss (several species, mainly *Pleurozium schreberi*) sampled in Austria (Σ16 PAH 81–150 ng g^−1^ BaP 0.7–4 ng g^−1^) [62]. They were 100–1000 times lower than PAH concentrations observed in *B. rutabulum (Hewd.) Schimp* at a densely populated and traffic-impacted area in NE Spain in 2011 [5] and 10–100 times lower than Σ17 PAH concentrations observed in moss (*Hylocomium splendens, Pleurozium schreberi*) at an urban site in Poland [10].

Except for Nap, Acy and Ace, all of the remaining target PAH were quantified in moss. The absence of the low molecular weight PAH in all samples may be a result of losses during the analytical process as the recovery rates of corresponding internal standards were quite low. The composition profiles were similar at all sites with Bb + jF, Fla, Pyr and Phe as dominant compounds (contribution of about 10–20%, each). This is similar to findings in wet-only deposition (precipitation only) at background sites of the German Environment Agency (Westerland, Zingst, Schauinsland, Waldhof, Schmücke) (UBA, pers. communication, 2016). In the same samples, proportions of Phe increased to 25–70% if the funnel rinse is included. In ambient air samples at the same monitoring sites, Phe contributions of 35–45% were observed (UBA, pers. communication, 2016). As reviewed by Harmens et al. [24], mosses appear to be most appropriate for measuring environmental chemicals which are particle-deposited (i.e. compounds of higher log K_OW_ and log K_OA_ values, also see below). This may also contribute to the underrepresented Phe fraction in moss compared to air or precipitation (including funnel rinse) and is corroborated by Foan et al. [19] reporting bioconcentration factors of PAH in mosses being significantly correlated with K_OW_ values.

#### PCDD/F

**Fig. 4:**
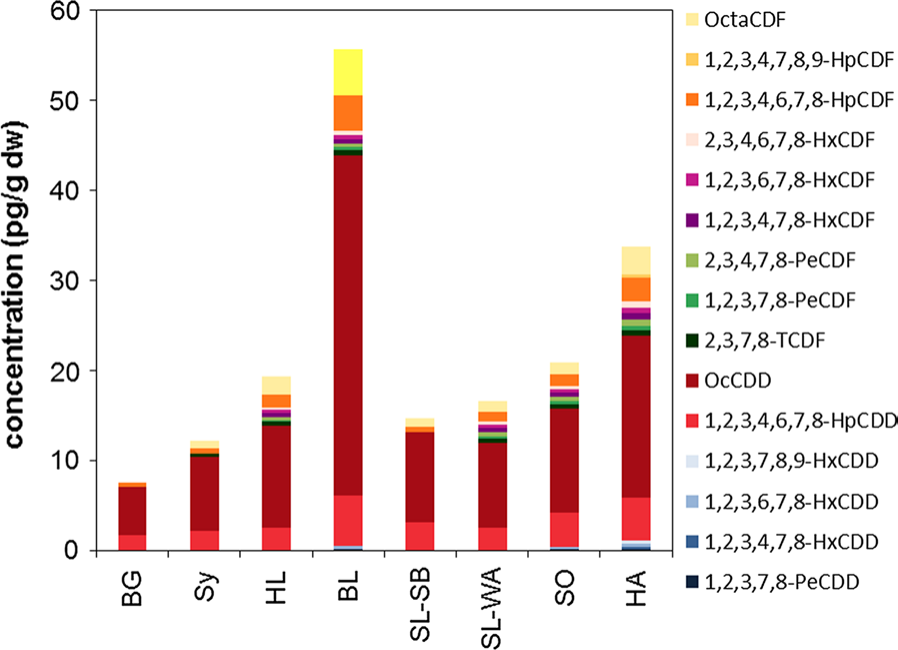
PCDD/F concentrations (pg g^−1^ dw) observed in German moss samples

Σ17PCDD/F concentrations were also lowest at the BG site (0.8 pg g^−1^ fw, 7.5 pg g^−1^ dw) and highest at the BL site in northern Germany (10 pg g^−1^ fw, 56 pg g^−1^ dw, Fig. 4, Additional file 1: Figure S2, Tables S7, S8). Taking toxicity into account, PCDD/F TEQ concentrations (without MQL) ranged from 0.024 pg TEQ_WHO2005_ g^−1^ dw (0.003 pg TEQ_WHO2005_ g^−1^ fw) to 0.81 pg TEQ_WHO2005_ g^−1^ dw (0.12 pg TEQ_WHO2005_ g^−1^ fw). PCDD/F have hardly been analysed in moss. Caraballeira et al. [7] reported PCDD/F concentrations of 10 pg g^−1^ dw or 0.3 pg TEQ g^−1^ dw (in woodlands) to 422 pg g^−1^ dw or 34 pg TEQ g^−1^ dw (close to a landfill) in *Pseudoscleropodium purum* from Spain. Recently, Danielsson et al. [8] observed PCDD/F concentrations in Swedish moss samples (*Pleurozium schreberi* or *Hylocomium splendens*) from 0.0001 to 0.57 pg TEQ_WHO2005_ g^−1^ dw. Thus, maximum TEQ-based PCDD/F moss concentrations were similarly high in Sweden and Germany; whereas, lowest TEQ values were about ten times lower in Sweden. PCDD/F have been analysed in lichens slightly more frequently, e.g. recently by Augusto et al. [3] in the Mediterranean region with average PCDD/F concentrations of 35 pg g^−1^ in 2011 to 150 pg g^−1^ in 2000.

With 55–70%, OCDD dominated the PCDD/F profiles followed by 1,2,3,4,6,7,8-HpCDD, OCDF, and 1,2,3,4,6,7,8-HpCDF. In contrast to samples SL-SB, BY and BG, where only these three congeners were quantified, remaining samples also comprised other PCDD/F congeners, however, at low concentrations close to the MQL. 2,3,7,8-TCDD was not detected in any sample. The rather similar PCDD/F profiles in moss suggest a widespread source-distant PCDD/F contamination at the investigated sites at slightly differing but low levels which resembled typical atmospheric background profiles [45].

#### dl-PCB

**Fig. 5:**
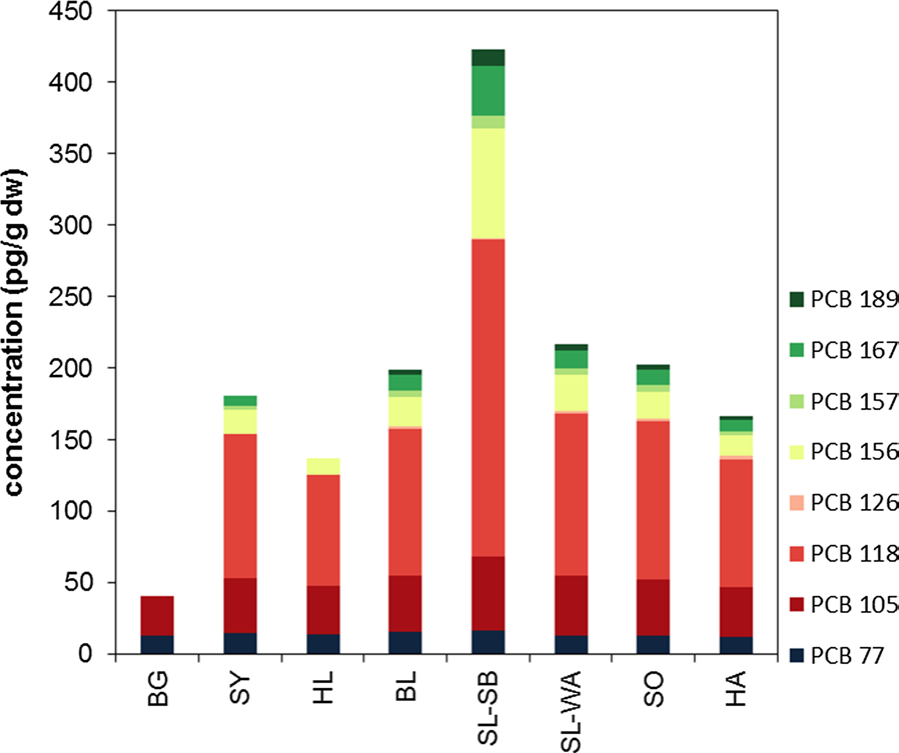
dl-PCB concentrations (pg g^−1^ dw) observed in German moss samples

Total concentrations of dioxin-like PCB in investigated moss samples varied by a factor of ten between the sites with a range from 41 pg g^−1^ dw (4.4 pg g^−1^ fw) to 423 pg g^−1^ dw (40.6 pg g^−1^ fw, Fig. 5, Additional file 1: Figure S3, Tables S9, S10). Concentrations were lowest at BG and highest at the Saarland site SL-SB. At the remaining sites, concentrations of dl-PCB did not differ much and were between 137 pg g^−1^ dw and 216 pg g^−1^ dw. Concentrations on the basis of TEQ (w/o MQL) were between 0.002 pg TEQ_WHO2005_ g^−1^ dw (BG) and 0.22 pg TEQ_WHO2005_ g^−1^ dw (HA). Except for SL-SB, TEQ values resulting from dl-PCB contamination were lower than those from PCDD/F contamination. Similar to PCDD/F, maximum dl-PCB concentrations in German moss samples were in the same order, minimum concentrations up to a factor of hundred higher than concentrations observed in recent investigations on moss (*Pleurozium schreberi* or *Hylocomium splendens*) in Sweden (0.00003–0.073 pg TEQ_WHO2005_ g^−1^ dw).

Except for BG where only two dl-PCB were quantified, dl-PCB profiles in moss samples were quite similar to PCB 118 as dominating compound (50–55%) followed by PCB 105 (12–25%), PCB 156 (9–18%) and PCB 77 (4–10%). At BG, only PCB 77 and PCB 105 were observed. The predominance of PCB 118 and elevated contributions of the other above-mentioned dl-PCB was also observed in German ambient air and deposition samples, although with higher PCB 118 proportions compared to moss samples [45].

#### ndl-PCB

In all but one sample which was located in the Saarland conurbation (SL-SB), ndl-PCB concentrations were below the MQL (Additional file 1: Tables S11, S12). At the SL-SB site, only three ndl-PCB (PCB 138, PCB 153, PCB 180) were quantified at individual concentrations ranging from 0.6 to 1 ng g^−1^ dw (0.06–0.1 ng g^−1^ fw). In general, method quantification limits of ndl-PCB are higher than those of dl-PCB due to the ubiquitous presence of ndl-PCB, also in laboratory blank samples. Therefore, dl-PCB have been quantified whereas ndl-PCB usually were not. Still, the low frequency of quantification was surprising, as marker PCB are common pollutants in German forest soils (up to 106 ng g^−1^ dw), particularly in western Germany [1]. Similar to our results, concentrations of marker PCB (0.19–0.84 ng g^−1^ dw, mainly PCB 101, PCB 153) were mostly close to or below the MQL in Swedish moss (*Pleurozium schreberi* or *Hylocomium splendens*) [8]. Levels for Σ25 PCB observed in moss (*Drepanocladus aduncus*) from Svalbard were in the same order (0.43–1.16 ng g^−1^) with Mono- to HexaCB being the dominant homologues [63]. Thüns [53] observed ndl-PCB in *Sphagnum*-dominated peat bogs of Eastern Canada; however, significant post depositional mobility of PCB on longer time scales was described which hampered the use of these peatlands as PCB deposition archives. Such mobility may have also influenced PCB accumulation in recent moss samples.

#### PFAS

PFAS were generally not quantified (MQL < 0.15 ng g^−1^ dw) in investigated moss samples with the exception of PFOS (0.2 ng g^−1^ dw; 0.04 ng g^−1^ fw) in the Bornhöved Lakes region. Similarly, Danielsson et al. [8] did not find PFAS in Swedish moss. PFAS have been found in precipitation in Germany and elsewhere, e.g. [11, 61] and due to the phase-out of long-chain PFAS a shift of composition to short-chain PFAS (among other compounds) was already noted in European environmental archives [30]. Therefore, it is not surprising that Falk et al. [16] observed mainly PFBA (1.5–5 ng g^−1^) in German tree leave samples. PFOA (< 0.07–0.2 ng g^−1^) and PFOS (< 0.07–0.2 ng g^−1^) were also often detected, however, at lower concentrations. Likewise, Yeung et al. [61] found mainly short-chain PFAS in recently taken rain water samples. However, exactly these short-chain PFAS have been found to be highly mobile and to be only little retained in soil or *Sphagnum*-dominated peatlands [13]. Thus, mosses do not appear to be good bioindicators for atmospheric PFAS pollution.

#### Flame retardants

**Fig. 6:**
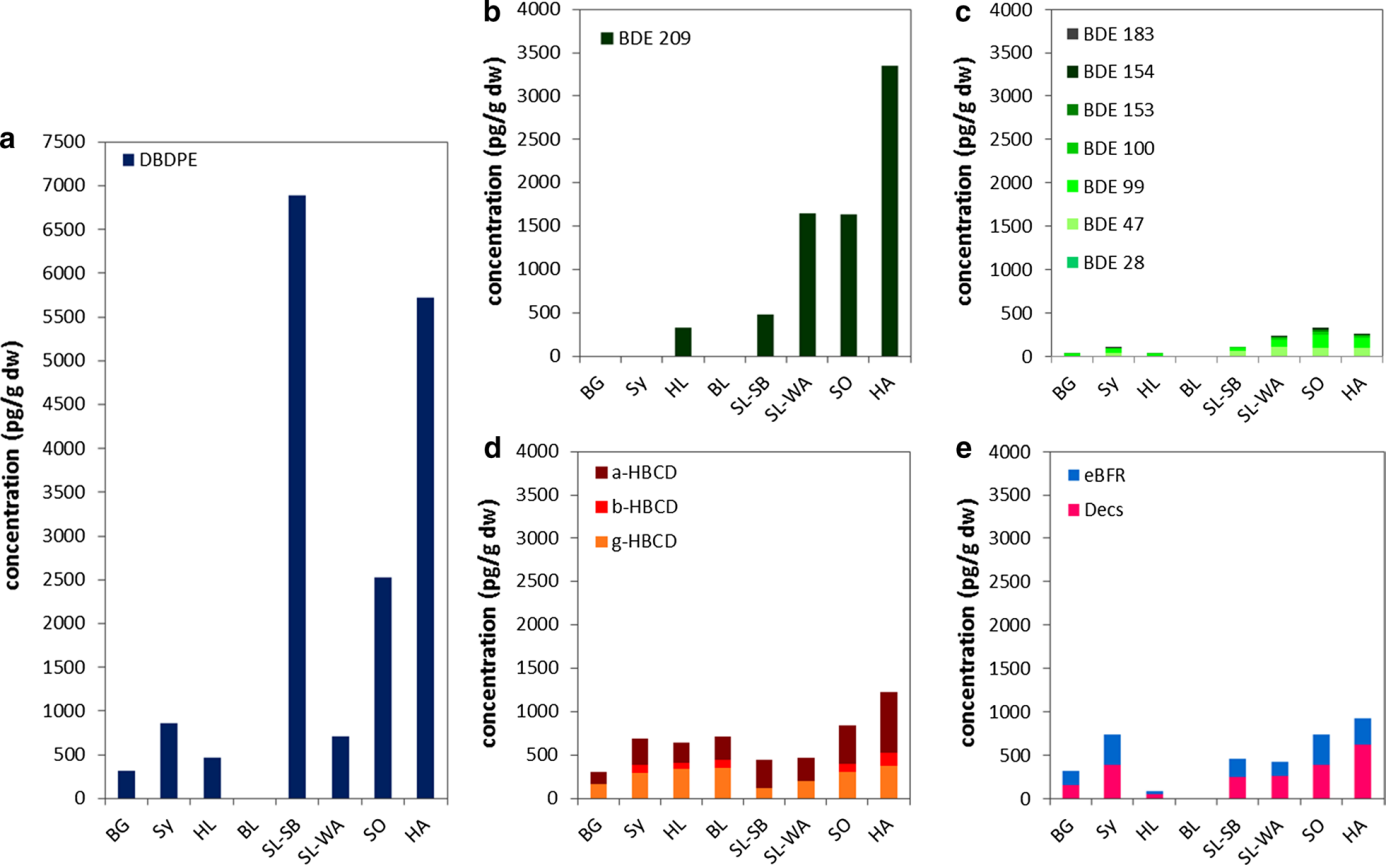
Concentrations (ng g^−1^ dw) of DBDPE (**a**), BDE 209 (**b**), Σ7BDE (**c**; sum of BDE 28, 47, 99, 100, 153, 154, 183), HBCD (**d**), and quantified emerging flame retardants [**e**; Dechloranes (Decs): sum of DP, Cl_11_anti-DP, Dec602; *emerging* BFR (eBFR): sum of ATE, BATE, DPTE, PBT, HBBz, PBEB] observed in German moss samples

Figure 6 shows concentrations of flame retardants. Individual graphs have been arranged to scale to facilitate comparisons. It becomes obvious that one single compound, DBDPE, dominates the investigated flame-retardant substance spectra. The DBDPE proportion was lowest in Bavarian samples (SY, BG) and highest at one Saarland site (SL-SB). Similar to DP, which was found to be much less prominent in German moss samples, DBDPE was reported to replace DecaBDE [47, 58], the second most dominant substance in the investigated samples. HBCD was observed at third highest concentrations. Compared to these three substances, remaining individual PBDE, PBB or *emerging* flame retardants were observed at concentrations being much lower or below the respective MQL (Additional file 1: Tables S15–S18, Figures S5–S7).

Concentrations of Σ23PBDE (i.e. w/o deca-BDE 209) were between 0.027 ng g^−1^ dw (0.003 ng^−1^ fw) at BG and 0.29 ng g^−1^ dw (0.043 ng g^−1^ fw) at HA. BDE 209 concentrations ranged from < 0.95 ng g^−1^ dw (< 0.1 ng g^−1^ fw) at BG to 3.35 ng g^−1^ dw (0.5 ng g^−1^ fw) at HA. Besides BDE 209, main BDE contributions were observed for BDE 99, BDE 47 and BDE 100. Other PBDE occasionally quantified close to the MQL were BDE 28, BDE 49, BDE 66, BDE 85, BDE 153, BDE 154, BDE 183, BDE 206 and BDE 207. Besides the strong DecaBDE signal, the pattern of remaining PBDE was similar to the congener pattern in a technical penta-mixture that is characterized by a dominance of BDE 99 and BDE 47 followed by minor contributions of BDE 100, BDE 153 and BDE 154 [32]. Of eight PBDE investigated in moss samples (*Pleurozium schreberi* or *Hylocomium splendens*) from Sweden, TetraBDE 47 was nearly exclusively observed at concentrations of 0.06–0.46 ng g^−1^ dw [8]. For Norwegian moss samples (*Hylocomium splendens*), Σ7PBDE concentrations of 0.03–0.34 ng g^−1^ dw and BDE 209 concentrations of 0.05–1.6 ng g^−1^ dw were published [32]. With a dominance of BDE 209 and major contributions of BDE 47 and BDE 99, PBDE profiles were similar to German moss samples. The presence of PBDE in mosses at very remote locations was shown by Wang et al. [59] (Σ12PBDE w/o BDE 209: 0.122 ng g^−1^ dw, mainly BDE 47, BDE 28 and BDE 99) and Zhu et al. [63] (Σ13PBDE w/o BDE 209: 0.119 ng g^−1^ dw, mainly BDE 47, BDE 99, BDE 183; *Drepanocladus aduncus*) at Svalbard, by Zhu et al. [64] at the Tibetan plateau (Σ13PBDE: 0.16 ng g^−1^ dw, mainly BDE 183; *Pottiaceae*, *Hypnaceae*) and by Kim et al. [29] at the south Shetland Islands, Antarctica (Σ19PBDE: 0.003–0.07 ng g^−1^ dw; mainly BDE 47, BDE 99; *Sanionia uncinata*, *Andreaea depressinervis*).

*Emerging* brominated flame retardants were quantified in all samples. DBDPE concentrations ranged from 0.3 ng g^−1^ dw (0.04 ng g^−1^ fw) at BG to 6.9 ng g^−1^ dw at SL-SB (1 ng g^−1^ fw at HA). Other eBFR quantified in almost all samples although at lower concentrations were DPTE (< 0.1 ng g^−1^ dw/< 0.015 ng g^−1^ fw at L to 0.26 ng g^−1^ dw at SY/0.04 ng g^−1^ fw at HA) as well as ATE, BATE, HBBz, and PBT. TBA was only occasionally observed. BTBPE, EHTeBB, BEHTBP, and PBEB were not observed in any sample. Without DBDPE, the composition of *emerging* brominated flame retardants was quite similar between the moss samples with major contributions of DPTE (Additional file 1: Figure S7). The dominance of DBDPE and major contributions of DPTE were also observed in tree leave samples of nearby sites [12]. Among other HFR, DBDPE was observed in moss samples taken at Faroes (0.14–0.34 ng g^−1^ dw; *Hylocomium splendens*, [47]). In contrast to our study, Schlabach et al. [47] also observed PBEB (0.004–0.006 ng g^−1^ dw) and BTBPE (0.06–0.15 ng g^−1^ dw) in these moss samples. Compared to German moss samples, concentrations of HBBz were in the same order, those of PBT by a factor of 10 lower.

Of chlorinated flame retardants, DP was observed in all samples (0.05 ng g^−1^ dw/0.01 ng g^−1^ fw at HL to 0.6 ng g^−1^ dw/0.1 ng g^−1^ fw at HA). Dec602 and the DP degradation product Cl_11_-antiDP were only occasionally found. Dec603, Dec604, DPMA and Cl_10_-antiDP were not quantified in any sample. DP concentrations in moss of the South Shetland Islands, Antarctica were between 0.001 and 2.4 ng g^−1^ dw (*Andreaea depressinervis, Sanionia uncinata*; [29]). These authors also quantified Dec602 at concentrations below 0.0025 ng g^−1^ dw. Na et al. [35] reported DP concentrations of 0.0014 ng g^−1^ dw in moss collected from Svalbard. The fraction of the anti-DP-isomer (*f*(anti) = [anti-DP]/[ΣDP]) is often used to investigate stereo selective enrichment or degradation of one or the other DP isomer because the final DP product contains syn-DP and anti-DP in a certain ratio (f(anti):0.6–0.8) [27, 51, 55]. In the German moss samples, *f*(anti) values were in the narrow range between 0.74 and 0.79 (0.76 on average) which does not indicate a significant selective enrichment. Moss *f*(anti) values were similar to those observed in tree leave samples from nearby sites (0.72 on average [12]) but showed less variance. They were higher than values reported for European air masses investigated in 2008 (*f*(anti) in sample English Canal: 0.63; [34]).

HBCD was also quantified in all moss samples. Similar to other contaminants, lowest concentrations were observed at BG (0.03 ng g^−1^ fw, 0.3 ng g^−1^ dw). Highest concentrations were found at HA (0.18 ng g^−1^ fw, 1.2 ng g^−1^ dw). Interestingly, HBCD concentrations in the Saarland conurbation (SL-SB, SL-WA) were rather low whereas concentrations for other flame retardants belonged to the highest that were observed (see below). In Sweden and Norway, HBCD was not detected in any moss sample [8]. Kim et al. [29] observed between 0.002 and 2.4 ng g^−1^ dw HBCD in mosses (*Andreaea depressinervis, Sanionia uncinata*) of South Shetland Islands, Antarctica. HBCD was also observed in lichen from the Tibetan plateau (0.14 ng g^−1^ dw) but not in moss [64]. All of the three HBCD isomers were observed in German moss samples. 35–73% of the HBCD composition was made of α-HBCD, 27–53% of γ-HCBD and up to 12% of β-HBCD. At sites of low HBCD concentrations, levels of β-HBCD were below the MQL. Thus, similar to findings in fauna [4], α-HBCD appeared to be preferentially accumulated in moss compared to technical mixtures, where its presence is only 3–30%. This is in contrast to findings of Zhu et al. [64] who reported an HCBD composition in lichen being close to that of technical mixtures.

### Sources

For the evaluation of pollutant sources, source regions or transport pathways correlation analyses, multivariate statistics and fingerprinting by comparing profiles or certain diagnostic ratios (DR) are often used. The low number of sampling sites/samples of the pilot study presented limits the applications and informative value of such approaches for our data set. Thus, within the Moss Survey 2020, the number of moss sampling sites for POP analyses should be increased based on respective statistics derived by the presented pilot study 2015.

Correlation analyses on analyte concentrations (Spearman, *p* < 0.05) show strong positive and significant relationships for most, particularly high molecular weight PAH which indicates similar sources, probably combustion processes (see below). With few rather scattered exceptions, no such relationships were observed between individual PAH and other compounds pointing at different sources or pathways as those of PAH. Individual PCDD/F concentrations were correlated significantly to each other as well as individual dl-PCB. However, no relationships were observed between individual PCDD/F and individual dl-PCB indicating different sources (e.g. combustion-related sources (PCDD/F and dl-PCB), pigments (dl-PCB, ndl-PCB), technical PCB mixtures (dl-PCB, ndl-PCB) [33]), different transport behaviour and/or ad/absorption characteristics of the compounds or just the limitations of the small data set. Only few significant correlations existed between individual PBDE or HFR. PentaBDE were correlated as well as the polybrominated ethers DPTE and BATE or highly brominated and chlorinated flame retardants DBDPE, DP or BDE209 (Additional file 2).

Quass et al. [45] statistically investigated PCDD/F deposition and ambient air data from Germany. PCDD/F profiles in moss were similar to a typical background site reference profile for PCDD/F in ambient air where OCDD dominates the profile (52%) followed by 1,2,3,4,6,7,8-HpCDD (19%), OCDF (5.2%), and 1,2,3,4,6,7,8-HpCDF (5.7%). A general background site reverence profile for deposition samples was not reported due to a higher site-specific variance of PCDD/F composition; however, individual investigated deposition samples were characterized by the above-mentioned main congeners with an OCDD dominance that was, similarly to moss samples of the present study, more pronounced than in air samples. Thus, PCDD/F in moss probably originated from atmospheric background PCDD/F contamination.

**Fig. 7:**
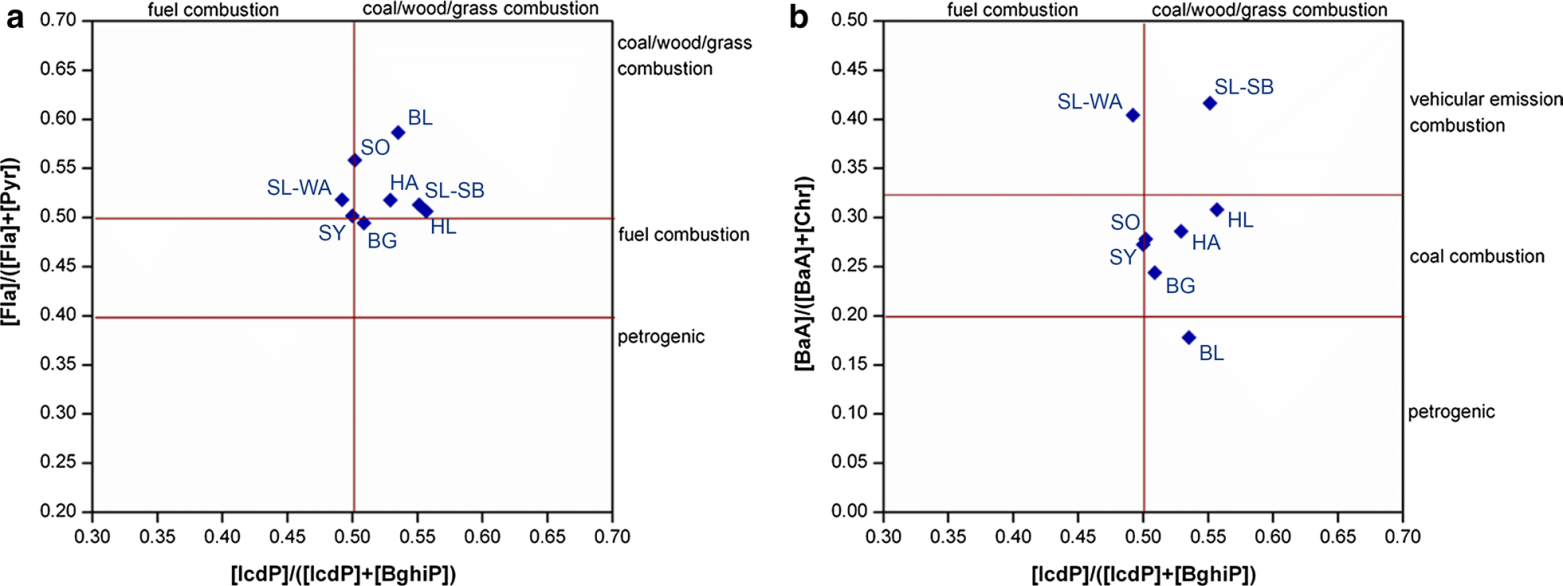
PAH cross plots for the diagnostic concentration ratios of **a** IcdP/(IcdP + BghiP) and Fla/(Fla + Pyr) and **b** IcdP/(IcdP + BghiP and BaA/(BaA + Chr). Diagnostic ratios and assignment of DR values from [54]

Diagnostic ratios of individual PAH or PAH groups are often used to distinguish different PAH sources, since PAH emission profiles depend on conditions during PAH generation [10, 14, 42, 54]. PAH diagnostic ratios have to be interpreted with caution. Depending on the physical chemical properties of individual PAH, their reactivity or (photo/oxidant) degradability, DR values may change during environmental transport or phase transitions [14, 54]. Dvorska et al. [14] concluded that DR are better predictors under conditions of limited availability of oxidants and when the reaction time (and thus distance) is short between source and receptor areas. Tobiszewski and Namiesnik [54] reported that the Fla/(Fla + Pyr) and IcdP/(IcdP + BghiP) ratios are more conservative than ratios of Ant/(Ant + Phe) and BaA/(BaA + Chr), which are more sensitive to photodegradation. Selected diagnostic ratios observed in German moss samples and their assignment to potential sources referring to Tobiszewski and Namiesnik [54] are depicted in Fig. 7 and Additional file 1: Table S23. According to these ratios, PAH in German mosses rather originated from mixed combustion processes. Uncertainties involved with this method become obvious looking at the DR for the BL site where two DR point at combustion-related PAH sources (Fla/(Fla + Pyr) = 0.59, IcdP/(IcdP + BghiP) = 0.54) whereas one points at petrogenic PAH sources (BaA/(BaA + Chr) = 0.18).

### Comparison of moss to tree leaf samples

Certain PAH and flame retardants were also determined in tree leave samples within the monitoring programme of the German ESB [12, 21]. As moss sampling was conducted close to some of the tree sampling sites, values of deciduous tree leaves (*Fagus sylvatica, Populus nigra*) and 1-year-old coniferous shoots (*Pinus sylvestris, Picea abies*) sampled in 2015 or 2016 can be compared to those observed in moss within the limitations due to the low number of samples.

In general, PAH and flame-retardant concentrations in moss, tree leaves and tree shoots were in the same order (Additional file 1: Figures S8, S9) showing that investigated mosses and tree leaves/shoots are generally suited biomonitors of atmospheric pollution of these contaminants. Few exceptions were observed for Phe, low molecular weight PAH at the conurbation sites HL, SL-WA and SL-SB, for TetraBDE and PentaBDE at HL and for DBDPE at SL-SB and HA. Individual PAH or flame-retardant concentrations in moss and tree leaves or shoots were not significantly correlated (Spearman, *p* < 0.05) which may have resulted from the small number of samples, different accumulation periods as well as small-scale differences regarding the locations of sampling sites. Accumulation of three-ring PAH, particularly Phe, increased in the order moss < deciduous leaves < coniferous shoots. In contrast, BaA, five- and six-ring PAH concentrations decreased in the order moss > deciduous leaves ≥ coniferous shoots. Observed differences for PAH were mostly significant (*U*-test, *p* < 0.05). For four-ring PAH, concentrations did not differ between moss and tree samples. These results are corroborated by findings of Oishi [42] who similarly described pine needles preferentially accumulating low molecular weight PAH. Except for syn-DP and anti-DP in moss and coniferous trees, HFR concentrations were not significantly different (*U*-test, *p* < 0.05).

**Fig. 8:**
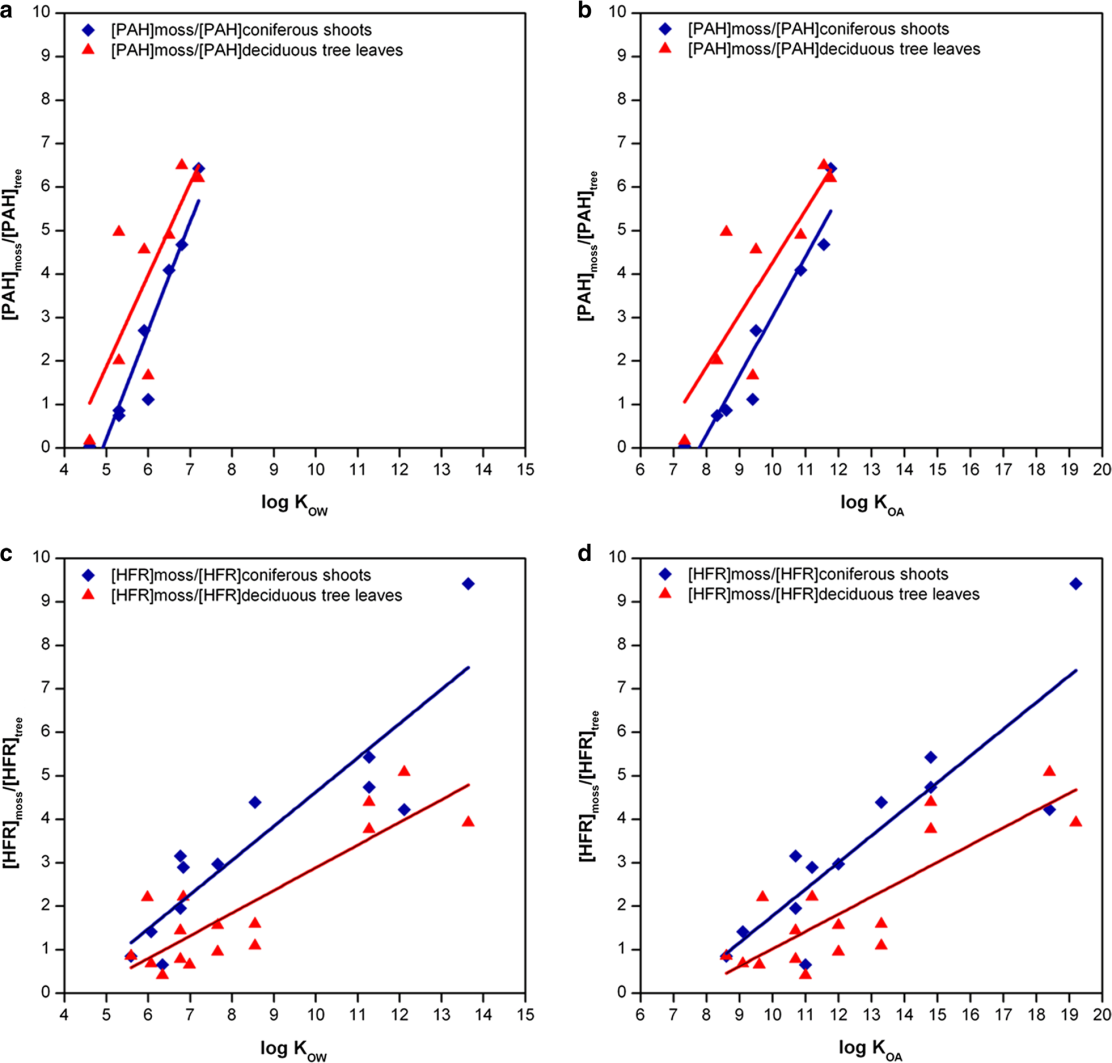
Averaged moss–tree leaf/shoot concentration ratios of PAH (**a**, **b**) and flame retardants (**c**, **d**) in relation to log K_OW_ and log K_OA_. PAH included were those PAH that had been analysed in moss samples as well as in coniferous shoot and deciduous leaf samples of the German ESB (Phe, Fla, Pyr, BaA, Chr, BaP, IcdP, BghiP). Log K_OW_ and log K_OA_ values for PAH from [41, 54], for flame retardants from [2, 23]

Differences expressed as ratios between concentrations observed in moss and concentrations observed in trees averaged over the investigated sites indicate a dependence on log K_OW_ and log K_OA_, i.e. the higher log K_OW_ or log K_OA_ values the more is accumulated in moss relative to tree leaves or shoots (Fig. 8). Observed correlations were significant (Spearman, *p* < 0.05). Similarly to PAH, average moss/tree concentration ratios for flame retardants increased with increasing log K_OW_ and log K_OA_ values (Fig. 8), although with flatter slopes and ratios being lower for deciduous trees than for conifers. This may be due to specific physical, chemical or morphological leaf characteristics, substance-specific properties, as well as emission properties with regard to the sampling time and/or accumulation period in the respective plant matrix. The exact mechanism for the substance class-related differences cannot be resolved for the present data set.

## Conclusion

Several organic contaminants (PAH, several PCDD/F, dl-PCB and flame retardants) were detected in moss sampled across Germany in the framework of a pilot study integrated into the European Moss Survey 2015. This pilot study shows the widespread spatial distribution as well as the suitability of moss as biomonitor for these pollutants. Limitations may, however, exist for the accumulation potential of PFAS. Compared to nearby tree leaf samples, accumulation potential of mosses appeared to be higher for pollutants of high log K_OA_ or K_OW_. Concentrations between the investigated sites did not differ by more than a factor of 10 (DBDPE 20). Lowest concentrations were usually observed at Berchtesgaden, the most pristine of investigated sites located in the German Alps. Elevated concentrations were often observed at the Saarland conurbation and the Harz, for PCDD/F and PAH also at the Bornhöved Lakes region, indicating potential source regions close to these areas. With the exception of dl-PCB and DBDPE at one Saarland site, contamination was not very pronounced at investigated conurbation areas. Given that quantified concentrations of some compounds were close to the respective MQLs and that MQLs, thus, had a strong influence on the compositions, substance class profiles were quite similar between the samples and indicated rather diffuse sources. Within the Moss Survey 2020, the number of moss sampling sites for POP analyses should be increased based on respective statistics derived by the presented pilot study 2015.

## Additional files

**Additional file 1.** Additional figures and tables.

**Additional file 2.** Spearman correlation.

## Abbreviations

BEHTeBP: bis(2-ethylhexyl)-tetrabromophthalate
DBDPE: decabromodiphenyl ethane
DecaBDE: decabromo diphenyl ether
dl-PCB: dioxin-like PCB
DP: Dechlorane Plus
eBFR: emerging brominated flame retardants
EHTeBB: 2-ethylhexyl-2,3,4,5-tetrabromobenzoate
ESB: Environmental Specimen Bank
GC-API–MS/MS: gas chromatography coupled to tandem mass spectrometry using atmospheric pressure ionization
GC–MS: gas chromatography coupled to mass spectrometry
HBB: hexabromobiphenyl
HBCD: hexabromocyclododecane
HFR: halogenated flame retardants
HPLC–ESI–MS/MS: high performance liquid chromatography using electrospray ionisation
HRGC–HRMS: high-resolution gas chromatography coupled to high-resolution mass spectrometry
Koa: octanol–air partition coefficient
Kow: octanol–water partition coefficient
MQL: method quantification limit
ndl-PCB: non dioxin-like PCB
PAH: polycyclic aromatic hydrocarbons
PBB: polybrominated biphenyls
PBDE: polybrominated diphenyl ether
PCB: polychlorinated biphenyls
PCDD/F: polychlorinated dibenzodioxins and -furans
PentaBDE: pentabromo diphenyl ether
PFAS: polyfluorinated alkyl substances
PFOS: perfluorooctane sulfonic acid
POP: persistent organic pollutants
