# Supplementary material for: (Persistent) Organic pollutants in Germany: results from a pilot study within the 2015 moss survey
Source: Environ Sci Eur. 2018 Nov 12;30(1):43. doi: 10.1186/s12302-018-0172-y (PMC6244560; doi:10.1186/s12302-018-0172-y)
Supplement: Supplementary file 1 — Additional file 1. Additional figures and tables. [file 12302_2018_172_MOESM1_ESM.pdf]

(Persistent) Organic Pollutants in Germany: results from a pilot study within the 2015 moss survey

Additional file

Annekatrin Dreyer[1*], Stefan Nickel[2], Winfried Schröder[3]

[1] Eurofins GfA GmbH, Air Monitoring, Stenzelring 14b, 21107 Hamburg, Germany, annekatrindreyer@eurofins.de

[2] University of Vechta, P.O.B. 1553, 49364 Vechta, Germany, stefan.nickel@uni-vechta.de

[3] University of Vechta, P.O.B. 1553, 49364 Vechta, Germany, winfried.schroeder@uni-vechta.de

* corresponding author

Table S 1: Information on sampling sites

abbreviation	lat	lon	elev	sampled moss species	general land use
BG	47.59541	12.92295	740	Pleurozium schreberi	near natural/background
HA	51.821196	10.639492	795	Pleurozium schreberi	near natural
SY	48.4807	11.43903	480	Hypnum cupressiforme	agrarian
BL	54.10608	10.2462	44	Pseudoscleropodium purum	agrarian
SO	51.757258	9.571325	485	Pleurozium schreberi	forstry
L	51.670097	12.589887	141	Pseudoscleropodium purum	conurbation
SL-SB	49.27898	6.97683	260	Pseudoscleropodium purum	conurbation
SL-WA	49.22595	6.78962	220	Pseudoscleropodium purum	conurbation

Table S 2: Target compounds, their abbreviations and method quantification limits

substance	abbreviation	MQL	unit
2,3,7,8-Tetrachlorodibenzo dioxin	2,3,7,8-TetraCDD	0.01	pg/g
1,2,3,7,8-Pentachlorodibenzo dioxin	1,2,3,7,8-PentaCDD	0.013	pg/g
1,2,3,4,7,8-Hexachlorodibenzo dioxin	1,2,3,4,7,8-HexaCDD	0.02	pg/g
1,2,3,6,7,8-Hexachlorodibenzo dioxin	1,2,3,6,7,8-HexaCDD	0.028	pg/g
1,2,3,7,8,9-Hexachlorodibenzo dioxin	1,2,3,7,8,9-HexaCDD	0.026	pg/g
1,2,3,4,6,7,8-Heptachlorodibenzo dioxin	1,2,3,4,6,7,8-HeptaCDD	0.043	pg/g
Octachlorodibenzo dioxin	OctaCDD	0.31	pg/g
2,3,7,8-Tetrachlorodibenzo furan	2,3,7,8-TetraCDF	0.028	pg/g
1,2,3,7,8-Pentachlorodibenzo furan	1,2,3,7,8-PentaCDF	0.019	pg/g
2,3,4,7,8-Pentachlorodibenzo furan	2,3,4,7,8-PentaCDF	0.03	pg/g
1,2,3,4,7,8-Hexachlorodibenzo furan	1,2,3,4,7,8-HexaCDF	0.032	pg/g
1,2,3,6,7,8-Hexachlorodibenzo furan	1,2,3,6,7,8-HexaCDF	0.029	pg/g
1,2,3,7,8,9-Hexachlorodibenzo furan	1,2,3,7,8,9-HexaCDF	0.021	pg/g
2,3,4,6,7,8-Hexachlorodibenzo furan	2,3,4,6,7,8-HexaCDF	0.026	pg/g
1,2,3,4,6,7,8-Heptachlorodibenzo furan	1,2,3,4,6,7,8-HeptaCDF	0.03	pg/g
1,2,3,4,7,8,9-Heptachlorodibenzo furan	1,2,3,4,7,8,9-HeptaCDF	0.021	pg/g
Octachlorodibenzo furan	OctaCDF	0.064	pg/g
3,3',4,4'-Tetrachlorobiphenyl	PCB 77	0.96	pg/g
3,4,4',5-Tetrachlorobiphenyl	PCB 81	0.14	pg/g
2,3,3',4,4'-Pentachlorobiphenyl	PCB 105	2.1	pg/g
2,3,4,4',5-Pentachlorobiphenyl	PCB 114	0.28	pg/g
2,3',4,4',5-Pentachlorobiphenyl	PCB 118	7.5	pg/g
2',3,4,4',5-Pentachlorobiphenyl	PCB 123	0.21	pg/g
3,3',4,4',5-Pentachlorobiphenyl	PCB 126	0.13	pg/g
2,3,3',4,4',5-Hexachlorobiphenyl	PCB 156	1.2	pg/g
2,3,3',4,4',5'-Hexachlorobiphenyl	PCB 157	0.22	pg/g
2,3',4,4',5,5'-Hexachlorobiphenyl	PCB 167	0.59	pg/g
3,3',4,4',5,5'-Hexachlorobiphenyl	PCB 169	0.64	pg/g
2,3,3',4,4',5,5'-Heptachlorobiphenyl	PCB 189	0.21	pg/g
2,4,4'-Trichlorobiphenyl	PCB 28	0.053	ng/g
2,2',5,5'-Tetrachlorobiphenyl	PCB 52	0.053	ng/g
2,2',4,5,5'-Pentachlorobiphenyl	PCB 101	0.053	ng/g
2,2',3,4,4',5'-Hexachlorobiphenyl	PCB 138	0.053	ng/g
2,2',4,4',5,5'-Hexachlorobiphenyl	PCB 153	0.053	ng/g
2,2',3,4,4',5,5'-Heptachlorobiphenyl	PCB 180	0.053	ng/g
2,2´,5,5´-Tetrabromobiphenyl	PBB 52	0.0022	ng/g
2,2´,4,5,5´-Pentabromobiphenyl	PBB 101	0.00331	ng/g
2,2´,4,4´,5,5´-Hexabromobiphenyl	PBB 153	0.00551	ng/g
2,2´,3,4,4´,5,5´-Heptabromobiphenyl	PBB 180	0.0331	ng/g
2,2´,3,3´,4,4´,5,5´-Octabromobiphenyl	PBB 194	0.0825	ng/g
2,2´,3,3´,4,4´,5,5´,6-Nonabromobiphenyl	PBB 206	0.11	ng/g
Decabromobiphenyl	PBB 209	0.22	ng/g

substance	abbreviation	MQL	unit
alpha-Hexabromocyclododecane	a-HBCD	0.00661	ng/g
beta-Hexabromocyclododecane	β-HBCD	0.00661	ng/g
gamma-Hexabromocyclododecane	γ-HBCD	0.00661	ng/g
Perfluoroctane sulfonic acid	PFOS	0.0105	ng/g
Perfluoroctanoic acid	PFOA	0.0105	ng/g
Perfluorbutane sulfonic acid	PFBS	0.0158	ng/g
Perfluorobutanoic acid	PFBA	0.0105	ng/g
Perfluoropentanoic acid	PFPA	0.0105	ng/g
Perfluorohexane suflonic acid	PFHxS	0.0158	ng/g
Perfluorohexanoic acid	PFHxA	0.0105	ng/g
Perfluoroheptane sulfonic acid	PFHpS	0.0158	ng/g
Perfluoroheptanoic acid	PFHpA	0.0105	ng/g
Perfluoroctane sulfonamide	PFOSA	0.0105	ng/g
Perfluorononanoic acid	PFNA	0.0105	ng/g
Perfluorodecane sulfonic acid	PFDS	0.0158	ng/g
Perfluorodecanoic acid	PFDA	0.0105	ng/g
Perfluoroundecanoic acid	PFUnA	0.0105	ng/g
Perfluorododecanoic acid	PFDoA	0.0105	ng/g
Perfluorotridecanoic acid	PFTrA	0.0105	ng/g
Perfluorotetradecanoic acid	PFTeA	0.0105	ng/g
Naphthalene	Nap	1.1	ng/g
Acenaphthylene	Ace	0.07	ng/g
Acenaphthene	Acy	0.09	ng/g
Fluorene	Flu	0.1	ng/g
Phenanthrene	Phe	0.28	ng/g
Anthracene	Ant	0.1	ng/g
Fluoranthene	Fla	0.1	ng/g
Pyrene	Pyr	0.1	ng/g
Benzo(a)anthracene	BaA	0.1	ng/g
Chrysene	Chr	0.1	ng/g
Benzo(b/j)fluoranthene (coelution)	BbjF	0.1	ng/g
Benzo(k)fluoranthene	BkF	0.1	ng/g
Benzo(a)pyrene	BaP	0.1	ng/g
Dibenz(ah)anthracene	DahA	0.1	ng/g
Indeno(1,2,3cd)pyrene	IcdP	0.1	ng/g
Benzo(ghi)perylene	BghiP	0.1	ng/g
2,4,6-Tribomoanisol	TBA	0.0425	ng/g
2,4,6-Tribromophenylallylether	ATE	0.013	ng/g
2-Bromoallyl-2,4,6-tribromophenylether	BATE	0.00425	ng/g
1,5-Dechloran Plus® Mono Addukt	DPMA	0.00025	ng/g
Pentabromotoluene	PBT	0.01075	ng/g
Pentabromoethylbenzene	PBEB	0.00075	ng/g
Hexabromobenzene	HBBz	0.0025	ng/g
2,3-Dibromopropyl-2,4,6-tribromophenylether	DPTE	0.0805	ng/g

substance	abbreviation	MQL	unit
Dechlorane 602	Dec602	0.00175	ng/g
2-Ethylhexyl-2,3,4,5-tetrabromobenzoate	EHTeBB	0.03325	ng/g
Dechlorane 603	Dec603	0.00075	ng/g
Dechlorane 604	Dec604	0.00125	ng/g
Cl10-Dechlorane Plus®	Cl10-AntiDP	0.0005	ng/g
1,2-Bis(2,4,6-tribromophenoxy)ethane	BTBPE	0.02475	ng/g
Bis(2-ethylhexyl)tetrabromophthalate	BEHTBP	0.115	ng/g
syn-Dechlorane Plus®	Syn-DP	0.006	ng/g
Cl11-Dechlorane Plus®	Cl11-AntiDP	0.00025	ng/g
anti-Dechlorane Plus®	Anti-DP	0.00675	ng/g
Decabromodiphenylethane	DBDPE	0.225	ng/g
2,2',4-Tribromodiphenyl ether	TriBDE (BDE-17)	0.0025	ng/g
2,4,4'-Tribromodiphenyl ether	TriBDE (BDE-28)	0.0025	ng/g
2,2',4,5'-Tetrabromodiphenyl ether	TetraBDE (BDE-49)	0.0025	ng/g
2,3',4',6-Tetrabromodiphenyl ether	TetraBDE (BDE-71)	0.0025	ng/g
2,2',4,4'-Tetrabromodiphenyl ether	TetraBDE (BDE-47)	0.03	ng/g
2,3',4,4'-Tetrabromodiphenyl ether	TetraBDE (BDE-66)	0.0025	ng/g
3,3',4,4'-Tetrabromodiphenyl ether	TetraBDE (BDE-77)	0.0025	ng/g
2,2',4,4',6-Pentabromodiphenyl ether	PentaBDE (BDE-100)	0.0025	ng/g
2,3',4,4',6-Pentabromodiphenyl ether	PentaBDE (BDE-119)	0.0025	ng/g
2,2',4,4',5-Pentabromodiphenyl ether	PentaBDE (BDE-99)	0.0125	ng/g
2,2',3,4,4'-Pentabromodiphenyl ether	PentaBDE (BDE-85)	0.0025	ng/g
3,3',4,4',5-Pentabromodiphenyl ether	PentaBDE (BDE-126)	0.0025	ng/g
2,2',4,4',5,6'-Hexabromodiphenyl ether	HexaBDE (BDE-154)	0.005	ng/g
2,2',4,4',5,5'-Hexabromodiphenyl ether	HexaBDE (BDE-153)	0.005	ng/g
2,2',3,4,4',5'-Hexabromodiphenyl ether	HexaBDE (BDE-138)	0.005	ng/g
2,3,3',4,4',5-Hexabromodiphenyl ether	HexaBDE (BDE-156)	0.005	ng/g
2,2',3,4,4',6,6'-Heptabromodiphenyl ether	HeptaBDE (BDE-184)	0.005	ng/g
2,2',3,4,4',5',6-Heptabromodiphenyl ether	HeptaBDE (BDE-183)	0.005	ng/g
2,3,3',4,4',5',6-Heptabromodiphenyl ether	HeptaBDE (BDE-191)	0.005	ng/g
2,2',3,3',4,4',6,6'-Octabromodiphenyl ether	OctaBDE (BDE-197)	0.025	ng/g
2,2',3,3',4,4',5,6'-Octabromodiphenyl ether	OctaBDE (BDE-196)	0.025	ng/g
2,2',3,3',4,4',5,6,6'-Nonabromodiphenyl ether	NonaBDE (BDE-207)	0.025	ng/g
2,2',3,3',4,4',5,5',6-Nonabromodiphenyl ether	NonaBDE (BDE-206)	0.025	ng/g
2,2',3,3',4,4',5,5',6,6'-Decabromodiphenyl ether	DecaBDE (BDE-209)	0.175	ng/g

Table S 3: Recovery rates (R, %) and standard deviations (SD, %) of mass-labelled internal standards (n=8)

substance	R (%)	SD (%)
$^{13}C_{12}$-2,3,7,8-TetraCDD	83	30
$^{13}C_{12}$-1,2,3,7,8-PentaCDD	90	31
$^{13}C_{12}$-1,2,3,4,7,8-HexaCDD	102	35
$^{13}C_{12}$-1,2,3,6,7,8-HexaCDD	101	35
$^{13}C_{12}$-1,2,3,4,6,7,8-HeptaCDD	78	27
$^{13}C_{12}$-OctaCDD	64	23
$^{13}C_{12}$-2,3,7,8-TetraCDF	83	29
$^{13}C_{12}$-1,2,3,7,8-PentaCDF	102	35
$^{13}C_{12}$-2,3,4,7,8-PentaCDF	87	30
$^{13}C_{12}$-1,2,3,4,7,8-HexaCDF	91	31
$^{13}C_{12}$-1,2,3,6,7,8-HexaCDF	100	34
$^{13}C_{12}$-1,2,3,7,8,9-HexaCDF	79	27
$^{13}C_{12}$-2,3,4,6,7,8-HexaCDF	95	32
$^{13}C_{12}$-1,2,3,4,6,7,8-HeptaCDF	92	32
$^{13}C_{12}$-1,2,3,4,7,8,9-HeptaCDF	74	26
$^{13}C_{12}$-OctaCDF	62	23
$^{13}C_{12}$-PCB 77	91	3
$^{13}C_{12}$-PCB 81	99	4
$^{13}C_{12}$-PCB 105	108	4
$^{13}C_{12}$-PCB 114	94	7
$^{13}C_{12}$-PCB 118	91	3
$^{13}C_{12}$-PCB 123	94	4
$^{13}C_{12}$-PCB 126	96	6
$^{13}C_{12}$-PCB 156	122	51
$^{13}C_{12}$-PCB 157	122	51
$^{13}C_{12}$-PCB 167	115	42
$^{13}C_{12}$-PCB 169	104	47
$^{13}C_{12}$-PCB 189	123	55
$^{13}C_{12}$-PCB 28	90	2
$^{13}C_{12}$-PCB 52	82	3
$^{13}C_{12}$-PCB 101	73	11
$^{13}C_{12}$-PCB 138	84	2
$^{13}C_{12}$-PCB 153	64	19
$^{13}C_{12}$-PCB 180	98	33
$^{13}C_{12}$-alpha-HBCD	79	9
$^{13}C_{12}$-gamma-HBCD	108	5
$^{13}C_{4}$-PFOS	158	91
$^{13}C_{2}$-PFDoA	11	8
$^{18}O_{2}$-PFHxS	147	80
$^{13}C_{2}$-M2PFTeDA	19	9
$^{13}C_{3}$-M3PFBS	170	109

**substance**	**R (%)**	**SD (%)**
$^{13}C_4$-PFBA	25	12
$^{13}C_2$-PFHxA	64	29
$^{18}O_2$-PFOA	58	13
$^{13}C_5$-PFNA	59	16
$^{13}C_2$-PFDA	44	12
$^{13}C_2$-PFUnA	33	19
Naphthalene-d_8	10	5
Acenaphthylene-d_8	31	6
Acenaphthene-d_8	18	3
Fluorene-d_{10}	31	3
Phenanthrene-d_{10}	55	4
Anthracene-d_{10}	53	4
Fluoranthene-d_{10}	64	5
Pyrene-d_{10}	59	5
Benzo(a)anthracene-d_{12}	82	6
Chrysene-d_{12}	73	5
Benzo(b)fluoranthene-d_{12}	80	8
Benzo(a)pyrene-d_{12}	77	5
Indeno(123-cd)pyrene-d_{12}	78	12
Benzo(ghi)perylene-d_{12}	77	9
Dibenz(ah)anthracene-d_{14}	81	17
^{13}C-HBBz	120	11
^{13}C-Dc602	113	7
^{13}C-EHTeBB	76	27
^{13}C-BTBPE	92	22
^{13}C-BEHTBP	29	28
^{13}C-SynDP	115	22
^{13}C-AntiDP	128	22
^{13}C-DBDPE	121	75
^{13}C-TriBDE (BDE-28)	106	31
^{13}C-TetraBDE (BDE-47)	119	32
^{13}C-PentaBDE (BDE-99)	108	24
^{13}C-HexaBDE (BDE-153)	109	16
^{13}C-HeptaBDE (BDE-183)	26	20
^{13}C-OctaBDE (BDE-197)	59	38
^{13}C-NonaBDE (BDE-207)	62	47
^{13}C-DecaBDE (BDE-209)	44	33

Table S 4: Results of analyses of certified reference materials

substance	unit	nominal value	measured value	deviation from nominal value
2,3,7,8-TetraCDD	pg/g	17 +- 3.9	23	6
1,2,3,7,8-PentaCDD	pg/g	3.71 +- 0.9	4.8	1.1
1,2,3,4,7,8-HexaCDD	pg/g	0.33 +- 0.18	0.4	0.1
1,2,3,6,7,8-HexaCDD	pg/g	2.03 +- 0.6	2.7	0.7
1,2,3,7,8,9-HexaCDD	pg/g	0.3 +- 0.14	< 0.47	-
1,2,3,4,6,7,8-HeptaCDD	pg/g	48 +- 0.36	< 0.76	-
OctaCDD	pg/g	1.71 +- 1.38	< 5.5	-
2,3,7,8-TetraCDF	pg/g	24.3 +- 4.74	31	6.7
1,2,3,7,8-PentaCDF	pg/g	4.58 +- 1.42	4.7	0.1
2,3,4,7,8-PentaCDF	pg/g	14.5 +- 4.04	18.4	3.9
1,2,3,4,7,8-HexaCDF	pg/g	5.95 +- 1.52	6.7	0.8
1,2,3,6,7,8-HexaCDF	pg/g	1.73 +- 0.54	2.3	0.5
1,2,3,7,8,9-HexaCDF	pg/g	0.1 +- 0.2	0.4	0.3
2,3,4,6,7,8-HexaCDF	pg/g	1.04 +- 0.3	1.5	0.4
1,2,3,4,6,7,8-HeptaCDF	pg/g	0.59 +- 0.44	1.4	0.8
1,2,3,4,7,8,9-HeptaCDF	pg/g	0.16 +- 0.32	0.6	0.5
OctaCDF	pg/g	0.38 +- 0.5	3.8	3.4
PCB 77	pg/g	1850 +- 834	2180	330
PCB 81	pg/g	161 +- 74	156	-5
PCB 105	pg/g	50100 +- 15660	51700	1600
PCB 114	pg/g	3410 +- 1548	3570	160
PCB 118	pg/g	122000 +- 38000	127000	5000
PCB 123	pg/g	3280 +- 2020	3680	400
PCB 126	pg/g	628 +- 242	746	118
PCB 156	pg/g	13100 +- 2720	12400	-700
PCB 157	pg/g	3380 +- 1012	3670	290
PCB 167	pg/g	7060 +- 3020	7510	450
PCB 169	pg/g	52.1 +- 14.02	57	5
PCB 189	pg/g	1440 +- 498	1530	90
PCB 28	ng/g	7.1 +- 1.26	9.2	2.1
PCB 52	ng/g	27.1 +- 12.1	31	4
PCB 101	ng/g	82.7 +- 21.4	84	2
PCB 138	ng/g	178 +- 27.8	169	-9
PCB 153	ng/g	226 +- 71.2	247	21
PCB 180	ng/g	108 +- 23.6	116	8
TriBDE (BDE-17)	ng/g		< 0.15	-
TriBDE (BDE-28)	ng/g	0.312 +- 0.2	0.33	0.02
TetraBDE (BDE-47)	ng/g	9.08 +- 2.62	9.2	0.1
TetraBDE (BDE-49)	ng/g	0.524 +- 0.27	0.54	0.01
TetraBDE (BDE-66)	ng/g	0.262 +- 0.08	< 0.30	-
TetraBDE (BDE-71)	ng/g		< 0.30	-
TetraBDE (BDE-77)	ng/g		< 0.30	-

substance	unit	nominal value	measured value	deviation from nominal value
PentaBDE (BDE-85)	ng/g		< 0.59	-
PentaBDE (BDE-99)	ng/g	2.28 +- 0.47	2.2	-0.1
PentaBDE (BDE-100)	ng/g	1.72 +- 0.57	1.6	-0.2
PentaBDE (BDE-119)	ng/g		< 0.59	-
PentaBDE (BDE-126)	ng/g		< 0.59	-
HexaBDE (BDE-138)	ng/g		< 0.89	-
HexaBDE (BDE-153)	ng/g	2.03 +- 0.51	1.8	-0.3
HexaBDE (BDE-154)	ng/g	2.55 +- 1	2.1	-0.5
HexaBDE (BDE-156)	ng/g		< 0.89	-
HeptaBDE (BDE-183)	ng/g	0.137 +- 0.05	< 1.5	-
HeptaBDE (BDE-184)	ng/g		< 1.5	-
HeptaBDE (BDE-191)	ng/g		< 1.5	-
OctaBDE (BDE-196)	ng/g		< 3.0	-
OctaBDE (BDE-197)	ng/g		< 3.0	-
NonaBDE (BDE-206)	ng/g		< 5.9	-
NonaBDE (BDE-207)	ng/g		< 5.9	-
DecaBDE (BDE-209)	ng/g	0.545 +- 2	< 16.2	-
Nap	ng/g		< 22.7	-
Acy	ng/g		< 0.36	-
Ace	ng/g		< 2.97	-
Flu	ng/g		< 2.46	-
Phe	ng/g		< 5.02	-
Ant	ng/g		< 0.12	-
Fla	ng/g		1	-
Pyr	ng/g		1	-
BaA	ng/g	3.64 +- 1.6	4.8	1.2
Chr	ng/g	5.06 +- 2.22	7	2
Bb+jF	ng/g	2.87 +- 1.26	3.7	0.8
BkF	ng/g	1.13 +- 0.5	1.3	0.2
BaP	ng/g	1.61 +- 0.71	2.2	0.5
DahA	ng/g	0.8 +- 0.35	0.9	0.1
IcdP	ng/g	1.19 +- 0.52	1.5	0.3
BghiP	ng/g	1.29 +- 0.57	1.5	0.2

Table S 5: Concentrations (ng/g dw) of PAHs in investigated moss samples

	BG	SY	L	BL	SL-SB	SL-WA	SO	HA
Nap	< 26.9	< 68	< 43.8	< 30.9	< 33.3	< 31.6	< 11.8	< 12.1
Acy	< 1.1	< 1.5	< 1	< 1.1	< 1.3	< 1.1	< 0.8	< 1.3
Ace	< 1	< 2.2	< 0.9	< 1.6	< 1.1	< 1.4	< 1	< 1.2
Flu	1.0	2.6	0.9	1.6	2.1	2.1	1.4	1.8
Phe	5.5	18.0	7.5	23.2	29.3	24.1	13.0	15.2
Ant	< 0.9	1.1	< 0.6	1.3	2.1	2.1	0.5	0.8
Fla	5.6	30.8	11.6	57.9	28.3	31.6	18.9	23.7
Pyr	5.7	30.5	11.3	40.7	26.9	29.4	14.9	22.1
BaA	1.2	5.5	4.0	9.5	14.1	10.0	3.9	7.2
Chr	3.6	14.5	8.9	43.9	19.7	14.7	10.1	17.9
Bb+jF	6.5	23.0	18.0	36.2	30.3	24.3	18.3	35.9
BkF	1.6	5.5	4.4	9.5	8.3	6.4	4.7	8.3
BaP	1.7	6.4	4.7	11.3	16.0	10.6	5.5	10.0
DahA	< 0.9	1.8	1.6	3.1	3.6	2.5	1.6	2.7
IcdP	3.5	11.3	9.6	15.8	19.6	10.7	9.3	18.2
BghiP	3.4	11.3	7.6	13.8	15.9	11.1	9.2	16.2
Σ16 PAH	39.3	162.3	90.1	267.8	216.2	179.6	111.3	180.0

Table S 6: Concentrations (ng/g fw) of PAHs in investigated moss samples

	BG	SY	L	BL	SL-SB	SL-WA	SO	HA
Nap	< 2.9	< 8.7	< 7.4	< 5.5	< 3.2	< 3.6	< 1.1	< 1.8
Acy	< 0.1	< 0.2	< 0.2	< 0.2	< 0.1	< 0.1	< 0.1	< 0.2
Ace	< 0.1	< 0.3	< 0.2	< 0.3	< 0.1	< 0.2	< 0.1	< 0.2
Flu	0.1	0.3	0.2	0.3	0.2	0.2	0.1	0.3
Phe	0.6	2.3	1.3	4.1	2.8	2.8	1.2	2.3
Ant	< 0.10	0.1	< 0.10	0.2	0.2	0.2	0.0	0.1
Fla	0.6	3.9	2.0	10.3	2.7	3.6	1.8	3.5
Pyr	0.6	3.9	1.9	7.3	2.6	3.4	1.4	3.3
BaA	0.1	0.7	0.7	1.7	1.4	1.1	0.4	1.1
Chr	0.4	1.9	1.5	7.8	1.9	1.7	0.9	2.7
Bb+jF	0.7	3.0	3.0	6.5	2.9	2.8	1.7	5.4
BkF	0.2	0.7	0.7	1.7	0.8	0.7	0.4	1.2
BaP	0.2	0.8	0.8	2.0	1.5	1.2	0.5	1.5
DahA	< 0.10	0.2	0.3	0.6	0.3	0.3	0.1	0.4
IcdP	0.4	1.4	1.6	2.8	1.9	1.2	0.9	2.7
BghiP	0.4	1.4	1.3	2.5	1.5	1.3	0.9	2.4
Σ16 PAH	4.2	20.8	15.2	**47.7**	20.8	20.5	10.4	26.8

Table S 7: Concentrations (pg/g dw) of PCDD/Fs in investigated moss samples

	BG	Sy	L	BL	SL-SB	SL-WA	SO	HA
2,3,7,8-TCDD	< 0.1019	< 0.1094	< 0.1006	< 0.1011	< 0.1042	< 0.1053	< 0.1019	< 0.1074
1,2,3,7,8-PeCDD	< 0.1296	< 0.1406	< 0.1302	0.159	< 0.1354	< 0.1316	0.146	0.146
1,2,3,4,7,8-HxCDD	< 0.2037	< 0.2188	< 0.2012	< 0.2022	< 0.2083	< 0.2018	< 0.2039	0.232
1,2,3,6,7,8-HxCDD	< 0.2778	< 0.2969	< 0.2722	0.368	< 0.2917	< 0.2807	0.343	0.422
1,2,3,7,8,9-HxCDD	< 0.2593	< 0.2813	< 0.2544	< 0.264	< 0.2708	< 0.2632	< 0.2575	0.303
1,2,3,4,6,7,8-HpCDD	1.787	2.211	2.633	5.674	3.188	2.535	3.723	4.779
OcCDD	5.287	8.281	11.302	37.753	9.938	9.474	11.588	18.054
2,3,7,8-TCDF	< 0.2778	0.304	0.381	0.516	< 0.2917	0.451	0.481	0.581
1,2,3,7,8-PeCDF	< 0.1944	< 0.2031	0.223	0.341	< 0.1979	0.286	0.333	0.463
2,3,4,7,8-PeCDF	< 0.2963	< 0.3203	0.313	0.455	< 0.3125	0.439	0.483	0.718
1,2,3,4,7,8-HxCDF	< 0.3148	< 0.3359	0.491	0.471	< 0.3333	0.460	0.442	0.678
1,2,3,6,7,8-HxCDF	< 0.2870	< 0.3047	0.305	0.406	< 0.3021	0.348	0.402	0.611
1,2,3,7,8,9-HxCDF	< 0.2130	< 0.2266	< 0.2071	< 0.2135	< 0.2188	< 0.2105	< 0.2146	< 0.2624
2,3,4,6,7,8-HxCDF	< 0.2593	< 0.2813	0.312	0.468	< 0.2708	0.332	0.391	0.698
1,2,3,4,6,7,8-HpCDF	0.494	0.606	1.349	4.028	0.698	1.105	1.298	2.664
1,2,3,4,7,8,9-HpCDF	< 0.2130	< 0.2188	< 0.2071	< 0.2079	< 0.2188	< 0.2105	< 0.2039	0.380
OctaCDF	< 0.6481	0.805	2.065	5.090	0.884	1.175	1.298	3.040
WHO(1998)-PCDD/F TEQ exkl. BG	0.023	0.060	0.358	0.730	0.040	0.431	0.662	0.960
WHO(1998)-PCDD/F TEQ inkl. 1/2 BG	0.328	0.368	0.521	0.815	0.356	0.597	0.748	1.027
WHO(1998)-PCDD/F TEQ inkl. BG	0.632	0.677	0.686	0.899	0.672	0.762	0.834	1.094
WHO(2005)-PCDD/F TEQ exkl. BG	0.024	0.061	0.294	0.640	0.042	0.339	0.561	0.812
WHO(2005)-PCDD/F TEQ inkl. 1/2 BG	0.297	0.336	0.456	0.725	0.325	0.505	0.647	0.879
WHO(2005)-PCDD/F TEQ inkl. BG	0.569	0.610	0.621	0.809	0.607	0.671	0.733	0.946
I-TEQ (NATO/CCMS) exkl. BG	0.028	0.068	0.370	0.685	0.050	0.440	0.600	0.906
I-TEQ (NATO/CCMS) inkl. 1/2 BG	0.299	0.341	0.500	0.775	0.330	0.573	0.686	0.973
I-TEQ (NATO/CCMS) inkl. BG	0.571	0.613	0.633	0.860	0.612	0.705	0.773	1.040

Table S 8: Concentrations (pg/g fw) of PCDD/Fs in investigated moss samples

	BG	Sy	L	BL	SL-SB	SL-WA	SO	HA
2,3,7,8-TCDD	< 0.011	< 0.014	< 0.017	< 0.018	< 0.010	< 0.012	< 0.0095	< 0.016
1,2,3,7,8-PeCDD	< 0.014	< 0.018	< 0.022	0.028	< 0.013	< 0.015	0.014	0.022
1,2,3,4,7,8-HxCDD	< 0.022	< 0.028	< 0.034	< 0.036	< 0.020	< 0.023	< 0.019	0.035
1,2,3,6,7,8-HxCDD	< 0.030	< 0.038	< 0.046	0.066	< 0.028	< 0.032	0.032	0.063
1,2,3,7,8,9-HxCDD	< 0.028	< 0.036	< 0.043	< 0.047	< 0.026	< 0.030	< 0.024	0.045
1,2,3,4,6,7,8-HpCDD	0.193	0.283	0.445	1.010	0.306	0.289	0.347	0.712
OcCDD	0.571	1.060	1.910	6.720	0.954	1.080	1.080	2.690
2,3,7,8-TCDF	< 0.030	0.039	0.064	0.092	< 0.028	0.051	0.045	0.087
1,2,3,7,8-PeCDF	< 0.021	< 0.026	0.038	0.061	< 0.019	0.033	0.031	0.069
2,3,4,7,8-PeCDF	< 0.032	< 0.041	0.053	0.081	< 0.030	0.050	0.045	0.107
1,2,3,4,7,8-HxCDF	< 0.034	< 0.043	0.083	0.084	< 0.032	0.052	0.041	0.101
1,2,3,6,7,8-HxCDF	< 0.031	< 0.039	0.052	0.072	< 0.029	0.040	0.038	0.091
1,2,3,7,8,9-HxCDF	< 0.023	< 0.029	< 0.035	< 0.038	< 0.021	< 0.024	< 0.020	< 0.0391
2,3,4,6,7,8-HxCDF	< 0.028	< 0.036	0.053	0.083	< 0.026	0.038	0.036	0.104
1,2,3,4,6,7,8-HpCDF	0.053	0.078	0.228	0.717	0.067	0.126	0.121	0.397
1,2,3,4,7,8,9-HpCDF	< 0.023	< 0.028	< 0.035	< 0.037	< 0.021	< 0.024	< 0.019	0.057
OctaCDF	< 0.070	0.103	0.349	0.906	0.085	0.134	0.121	0.453
WHO(1998)-PCDD/F TEQ exkl. BG	0.003	0.008	0.061	0.130	0.004	0.049	0.062	0.143
WHO(1998)-PCDD/F TEQ inkl. 1/2 BG	0.035	0.047	0.088	0.145	0.034	0.068	0.070	0.153
WHO(1998)-PCDD/F TEQ inkl. BG	0.068	0.087	0.116	0.160	0.065	0.087	0.078	0.163
WHO(2005)-PCDD/F TEQ exkl. BG	0.003	0.008	0.050	0.114	0.004	0.039	0.052	0.121
WHO(2005)-PCDD/F TEQ inkl. 1/2 BG	0.032	0.043	0.077	0.129	0.031	0.058	0.060	0.131
WHO(2005)-PCDD/F TEQ inkl. BG	0.062	0.078	0.105	0.144	0.058	0.077	0.068	0.141
I-TEQ (NATO/CCMS) exkl. BG	0.003	0.009	0.063	0.122	0.005	0.050	0.056	0.135
I-TEQ (NATO/CCMS) inkl. 1/2 BG	0.032	0.044	0.085	0.138	0.032	0.065	0.064	0.145
I-TEQ (NATO/CCMS) inkl. BG	0.062	0.079	0.107	0.153	0.059	0.080	0.072	0.155

Table S 9: Concentrations (pg/g dw) of dl-PCBs in investigated moss samples

	BG	SY	L	BL	SL-SB	SL-WA	SO	HA
PCB 77	12.870	15.313	13.787	15.562	16.563	12.983	13.090	12.013
PCB 81	< 1.4815	< 1.5625	< 1.4201	< 1.4607	< 1.4583	< 1.4035	< 1.3948	< 1.5436
PCB 105	28.056	38.203	34.083	39.832	52.292	41.667	39.163	35.235
PCB 114	< 2.8704	< 3.0469	< 2.7811	< 2.8652	< 2.9167	< 2.807	< 2.7897	< 3.0201
PCB 118	< 75.00	100.781	77.515	102.247	220.833	114.035	110.515	89.262
PCB 123	< 2.1296	< 2.2656	< 2.071	< 2.1348	< 2.1875	< 2.1053	< 2.1459	< 2.2819
PCB 126	< 1.2963	< 1.4063	< 1.3018	1.927	1.635	1.667	1.770	2.107
PCB 156	< 12.037	16.797	11.598	20.955	76.771	25.000	19.528	14.631
PCB 157	< 2.2222	2.383	< 2.1302	3.938	8.646	4.518	3.938	3.161
PCB 167	< 5.9259	7.773	< 5.7396	10.674	35.208	12.281	11.266	7.852
PCB 169	< 6.4815	< 6.7969	< 6.5089	< 6.1798	< 6.6667	< 6.4035	< 6.4378	< 6.7114
PCB 189	< 2.1296	< 2.2656	< 2.071	3.388	11.354	4.272	2.951	2.537
WHO(1998)-PCB TEQ exkl. BG	0.004	0.025	0.018	0.221	0.238	0.199	0.205	0.234
WHO(1998)-PCB TEQ inkl. 1/2 BG	0.112	0.131	0.117	0.255	0.272	0.232	0.238	0.269
WHO(1998)-PCB TEQ inkl. BG	0.219	0.238	0.215	0.288	0.306	0.265	0.272	0.304
WHO(2005)-PCB TEQ exkl. BG	0.002	0.007	0.005	0.199	0.178	0.174	0.184	0.217
WHO(2005)-PCB TEQ inkl. 1/2 BG	0.168	0.181	0.165	0.297	0.278	0.270	0.280	0.320
WHO(2005)-PCB TEQ inkl. BG	0.333	0.355	0.325	0.394	0.379	0.367	0.378	0.422

Table S 10: Concentrations (pg/g fw) of dl-PCBs in investigated moss samples

	BG	SY	L	BL	SL-SB	SL-WA	SO	HA
PCB 77	1.390	1.960	2.330	2.770	1.590	1.480	1.220	1.790
PCB 81	< 0.16	< 0.20	< 0.24	< 0.26	< 0.14	< 0.16	< 0.13	< 0.23
PCB 105	3.030	4.890	5.760	7.090	5.020	4.750	3.650	5.250
PCB 114	< 0.31	< 0.39	< 0.47	< 0.51	< 0.28	< 0.32	< 0.26	< 0.45
PCB 118	< 8.1	12.900	13.100	18.200	21.200	13.000	10.300	13.300
PCB 123	< 0.23	< 0.29	< 0.35	< 0.38	< 0.21	< 0.24	< 0.20	< 0.34
PCB 126	< 0.14	< 0.18	< 0.22	0.343	0.157	0.190	0.165	0.314
PCB 156	< 1.3	2.150	1.960	3.730	7.370	2.850	1.820	2.180
PCB 157	< 0.24	0.305	< 0.36	0.701	0.830	0.515	0.367	0.471
PCB 167	< 0.64	0.995	< 0.97	1.900	3.380	1.400	1.050	1.170
PCB 169	< 0.70	< 0.87	< 1.1	< 1.1	< 0.64	< 0.73	< 0.60	< 1.0
PCB 189	< 0.23	< 0.29	< 0.35	0.603	1.090	0.487	0.275	0.378
WHO(1998)-PCB TEQ exkl. BG	0.000	0.003	0.003	0.039	0.023	0.023	0.019	0.035
WHO(1998)-PCB TEQ inkl. 1/2 BG	0.012	0.017	0.020	0.045	0.026	0.026	0.022	0.040
WHO(1998)-PCB TEQ inkl. BG	0.024	0.031	0.036	0.051	0.029	0.030	0.025	0.045
WHO(2005)-PCB TEQ exkl. BG	0.000	0.001	0.001	0.036	0.017	0.020	0.017	0.032
WHO(2005)-PCB TEQ inkl. 1/2 BG	0.018	0.023	0.028	0.053	0.027	0.031	0.026	0.048
WHO(2005)-PCB TEQ inkl. BG	0.036	0.045	0.055	0.070	0.036	0.042	0.035	0.063

Table S 11: Concentrations (ng/g dw) of ndl-PCBs in investigated moss samples

	BG	SY	L	BL	SL-SB	SL-WA	SO	HA
PCB 28	< 0.537	< 0.5703	< 0.5207	< 0.5393	< 0.5521	< 0.5351	< 0.5365	< 0.5705
PCB 52	< 0.537	< 0.5703	< 0.5207	< 0.5393	< 0.5521	< 0.5351	< 0.5365	< 0.5705
PCB 101	< 0.537	< 0.5703	< 0.5207	< 0.5393	< 0.5521	< 0.5351	< 0.5365	< 0.5705
PCB 138	< 0.537	< 0.5703	< 0.5207	< 0.5393	0.791	< 0.5351	< 0.5365	< 0.5705
PCB 153	< 0.537	< 0.5703	< 0.5207	< 0.5393	0.996	< 0.5351	< 0.5365	< 0.5705
PCB 180	< 0.537	< 0.5703	< 0.5207	< 0.5393	0.580	< 0.5351	< 0.5365	< 0.5705

Table S 12: Concentrations (ng/g fw) of ndl-PCBs in investigated moss samples

	BG	SY	L	BL	SL-SB	SL-WA	SO	HA
PCB 28	< 0.058	< 0.073	< 0.088	< 0.096	< 0.053	< 0.061	< 0.050	< 0.085
PCB 52	< 0.058	< 0.073	< 0.088	< 0.096	< 0.053	< 0.061	< 0.050	< 0.085
PCB 101	< 0.058	< 0.073	< 0.088	< 0.096	< 0.053	< 0.061	< 0.050	< 0.085
PCB 138	< 0.058	< 0.073	< 0.088	< 0.096	0.076	< 0.061	< 0.050	< 0.085
PCB 153	< 0.058	< 0.073	< 0.088	< 0.096	0.096	< 0.061	< 0.050	< 0.085
PCB 180	< 0.058	< 0.073	< 0.088	< 0.096	0.056	< 0.061	< 0.050	< 0.085

Table S 13: Concentrations (ng/g dw) of HBCD in investigated moss samples

	BG	SY	L	BL	SL-SB	SL-WA	SO	HA
α-HBCD	0.142	0.311	0.226	0.275	0.323	0.272	0.440	0.698
β-HBCD	< 0.0628	0.084	0.067	0.087	< 0.0689	< 0.0643	0.098	0.156
γ-HBCD	0.160	0.298	0.345	0.353	0.119	0.196	0.305	0.377
Σ HBCD	0.302	0.694	0.639	0.714	0.441	0.467	0.842	1.228

Table S 14: Concentrations (ng/g fw) of HBCD in investigated moss samples

	BG	SY	L	BL	SL-SB	SL-WA	SO	HA
α-HBCD	0.015	0.040	0.038	0.049	0.031	0.031	0.041	0.104
β-HBCD	< 0.0068	0.011	0.011	0.016	< 0.0066	< 0.0073	0.009	0.023
γ-HBCD	0.017	0.038	0.058	0.063	0.011	0.022	0.028	0.056
Σ HBCD	0.033	0.089	0.108	0.127	0.042	0.053	0.079	0.183

Table S 15: Concentrations (pg/g dw) of PBDEs in investigated moss samples

	BG	SY	L	BL	SL-SB	SL-WA	SO	HA
BDE 17	< 2.7	< 2.9	< 2.7	n.a.	< 2.9	< 2.7	< 2.7	< 2.9
BDE 28	< 2.7	< 2.9	< 2.7	n.a.	< 2.9	< 2.7	3.0	< 2.9
BDE 49	< 2.7	< 2.9	< 2.7	n.a.	< 2.9	3.7	7.8	9.8
BDE 71	< 2.7	< 2.9	< 2.7	n.a.	< 2.9	< 2.7	< 2.7	< 2.9
BDE 47	< 32.4	40.6	< 32.4	n.a.	56.1	104.0	92.3	91.0
BDE 66	< 2.7	< 2.9	< 2.7	n.a.	< 2.9	< 2.7	5.0	7.1
BDE 77	< 2.7	< 2.9	< 2.7	n.a.	< 2.9	< 2.7	< 2.7	< 2.9
BDE 100	6.6	12.4	5.4	n.a.	14.2	26.4	39.7	24.7
BDE 119	< 2.7	< 2.9	< 2.7	n.a.	< 2.9	< 2.7	< 2.7	< 2.9
BDE 99	20.5	42.5	19.4	n.a.	37.8	84.9	150.0	121.0
BDE 85	< 2.7	< 2.9	< 2.7	n.a.	< 2.9	3.9	6.4	7.4
BDE 126	< 2.7	< 2.9	< 2.7	n.a.	< 2.9	< 2.7	< 2.7	< 2.9
BDE 154	< 5.4	< 5.8	< 5.4	n.a.	< 5.8	8.3	21.1	12.3
BDE 153	< 5.4	< 6.6	< 5.4	n.a.	< 5.8	13.2	21.6	12.0
BDE 138	< 5.4	< 5.8	< 5.4	n.a.	< 5.8	< 5.4	< 5.5	< 13.9
BDE 156	< 7.4	< 5.8	< 9.9	n.a.	< 10.5	< 5.4	< 10.1	< 29.2
BDE 184	< 110	< 5.8	< 35.2	n.a.	< 112	< 31.8	< 228	< 580
BDE 183	< 114	13.500	< 36.5	n.a.	< 116	< 32.9	< 236	< 601
BDE 191	< 115	< 5.8	< 37	n.a.	< 117	< 33.4	< 239	< 609
BDE 197	< 165	< 215	< 27	n.a.	< 2360	< 27	< 72.3	< 1700
BDE 196	< 175	< 228	< 27	n.a.	< 2130	< 27	< 65.3	< 1160
BDE 207	< 501	< 4020	< 27	n.a.	< 45.2	38.2	< 135	< 66
BDE 206	< 683	< 5480	< 27	n.a.	< 54.8	28.2	< 163	< 41.3
BDE 209	< 956	< 1150	324.0	n.a.	480.0	1650.0	1640.0	3350.0
Σ23PBDE	27.1	109.0	24.8	n.a.	108.1	310.8	346.9	285.3
Σ24PBDE	27.1	109.0	348.8	n.a.	588.1	1960.8	1986.9	3635.3

Table S 16: Concentrations (pg/g fw) of PBDEs in investigated moss samples

	BG	SY	L	BL	SL-SB	SL-WA	SO	HA
BDE 17	< 0.3	< 0.4	< 0.5	n.a.	< 0.3	< 0.3	< 0.3	< 0.4
BDE 28	< 0.3	< 0.4	< 0.5	n.a.	< 0.3	< 0.3	0.3	< 0.4
BDE 49	< 0.3	< 0.4	< 0.5	n.a.	< 0.3	0.4	0.7	1.5
BDE 71	< 0.3	< 0.4	< 0.5	n.a.	< 0.3	< 0.3	< 0.3	< 0.4
BDE 47	< 3.5	5.2	< 5.5	n.a.	5.4	11.9	8.6	13.6
BDE 66	< 0.3	< 0.4	< 0.5	n.a.	< 0.3	< 0.3	0.5	1.1
BDE 77	< 0.3	< 0.4	< 0.5	n.a.	< 0.3	< 0.3	< 0.3	< 0.4
BDE 100	0.7	1.6	0.9	n.a.	1.4	3.0	3.7	3.7
BDE 119	< 0.3	< 0.4	< 0.5	n.a.	< 0.3	< 0.3	< 0.3	< 0.4
BDE 99	2.2	5.5	3.3	n.a.	3.6	9.7	14.0	18.0
BDE 85	< 0.3	< 0.4	< 0.5	n.a.	< 0.3	0.4	0.6	1.1
BDE 126	< 0.3	< 0.4	< 0.5	n.a.	< 0.3	< 0.3	< 0.3	< 0.4
BDE 154	< 0.6	< 0.7	< 0.9	n.a.	< 0.6	0.9	2.0	1.8
BDE 153	< 0.6	< 0.8	< 0.9	n.a.	< 0.6	1.5	2.0	1.8
BDE 138	< 0.6	< 0.7	< 0.9	n.a.	< 0.6	< 0.6	< 0.5	< 2.1
BDE 156	< 0.8	< 0.7	< 1.7	n.a.	< 1	< 0.6	< 0.9	< 4.4
BDE 184	< 25.4	< 0.7	< 5.9	n.a.	< 18.9	< 3.6	< 16	< 48.6
BDE 183	< 25	1.7	< 6.2	n.a.	< 18.6	< 3.8	< 15.8	< 47.9
BDE 191	< 25.3	< 0.7	< 6.2	n.a.	< 18.9	< 3.8	< 15.9	< 48.5
BDE 197	< 17.8	< 27.6	< 4.6	n.a.	< 4.1	< 3.1	< 6.7	< 9.2
BDE 196	< 18.8	< 29.3	< 4.6	n.a.	< 4.4	< 3.1	< 6.1	< 9.7
BDE 207	< 53.9	< 515	< 4.6	n.a.	< 4.3	4.4	< 12.6	< 66.5
BDE 206	< 73.4	< 703	< 4.6	n.a.	< 5.3	3.2	< 15.2	< 90.6
BDE 209	< 103	< 148	54.6	n.a.	46.0	188.0	109.0	500.0
Σ23PBDE	2.9	14.0	4.2	n.a.	10.4	35.4	32.4	42.6
Σ24PBDE	2.9	14.0	58.8	n.a.	56.4	223.4	141.4	542.6

Table S 17: Concentrations (pg/g dw) of alternative flame retardants in investigated moss samples

	BG	SY	L	BL	SL-SB	SL-WA	SO	HA
TBA	< 42.5	< 42.5	< 42.6	n.a.	< 42.5	< 42.5	< 42.4	43.5
ATE	17.6	31.1	< 13	n.a.	13.8	13.1	19.5	13.8
BATE	17.2	32.9	15.4	n.a.	32.5	20.4	22.9	34.3
DPTE	100.0	263.0	< 80.7	n.a.	147.0	107.0	227.0	233.0
BTBPE	< 24.8	< 24.8	< 24.8	n.a.	< 24.8	< 24.8	< 24.7	< 40.1
EHTeBB	< 33.3	< 33.3	< 33.3	n.a.	< 33.3	< 33.3	< 33.2	< 33.2
BEHTBP	< 115	< 1100	< 115	n.a.	< 115	< 115	< 583	< 115
PBT	19.2	14.2	15.8	n.a.	14.9	15.7	36.8	14.3
HBBz	< 2.5	3.6	3.0	n.a.	< 2.5	2.8	35.6	3.4
PBEB	< 0.8	< 0.8	< 0.8	n.a.	< 0.8	< 0.8	< 0.7	< 0.7
DBDPE	313.0	861.0	463.0	n.a.	6890.0	712.0	2520.0	5720.0
Dec602	< 2.1	< 2.2	< 1.8	n.a.	< 3.3	10.0	< 1.8	3.7
Dec603	< 0.8	< 0.8	< 0.8	n.a.	< 0.8	< 0.8	< 0.7	< 0.7
Dec604	< 1.3	< 1.3	< 1.3	n.a.	< 1.3	< 1.3	< 1.3	< 2
DPMA	< 0.3	< 0.3	< 0.3	n.a.	< 0.3	< 0.3	< 1.3	< 0.3
Cl10-AntiDP	< 0.5	< 0.5	< 0.5	n.a.	< 0.5	< 0.5	< 0.8	< 0.7
Cl11-AntiDP	< 4.1	7.4	< 1.3	n.a.	< 4.4	5.3	6.0	12.0
Syn-DP	39.	99.7	12.3	n.a.	64.9	54.8	82.1	159.0
Anti-DP	117.	281.0	41.6	n.a.	181.0	188.0	303.0	451.0
ΣDP	156.6	380.7	53.9	n.a.	245.9	242.8	385.1	610.0
ΣHFR	623.6	1593.9	551.1	n.a.	7344.1	1129.1	3252.9	6688.0

Table S 18: Concentrations (pg/g dw) of alternative flame retardants in investigated moss samples

	BG	SY	L	BL	SL-SB	SL-WA	SO	HA
TBA	< 4.9	< 6.3	< 7.7	n.a.	< 4.7	< 5.2	< 4.3	7.5
ATE	2.0	4.6	< 2.4	n.a.	1.5	1.6	2.0	2.4
BATE	2.0	4.9	2.8	n.a.	3.6	2.5	2.3	5.9
DPTE	11.7	39.0	< 14.6	n.a.	16.3	13.2	23.1	40.2
BTBPE	< 2.9	< 3.7	< 4.5	n.a.	< 2.7	< 3.1	< 2.5	< 6.9
EHTeBB	< 3.9	< 4.9	< 6.1	n.a.	< 3.7	< 4.1	< 3.4	< 5.7
BEHTBP	< 13.3	< 162	< 20.9	n.a.	< 12.7	< 14.2	< 59.3	< 19.8
PBT	2.2	2.1	2.9	n.a.	1.7	1.9	3.8	2.5
HBBz	< 0.3	0.5	0.5	n.a.	< 0.3	0.3	3.6	0.6
PBEB	< 0.1	< 0.1	< 0.1	n.a.	< 0.1	< 0.1	< 0.1	< 0.1
DBDPE	36.2	127.0	83.9	n.a.	762.0	87.8	257.0	986.0
Dec602	< 0.2	< 0.3	< 0.3	n.a.	< 0.4	1.2	< 0.2	0.6
Dec603	< 0.1	< 0.1	< 0.1	n.a.	< 0.1	< 0.1	< 0.1	< 0.1
Dec604	< 0.1	< 0.2	< 0.2	n.a.	< 0.1	< 0.2	< 0.1	< 0.3
DPMA	< 0.1	< 0.1	< 0.1	n.a.	< 0.1	< 0.1	< 0.1	< 0.1
Cl10-AntiDP	< 0.1	< 0.1	< 0.1	n.a.	< 0.1	< 0.1	< 0.1	< 0.1
Cl11-AntiDP	< 0.5	1.1	< 0.2	n.a.	< 0.5	0.7	0.6	2.1
Syn-DP	4.6	14.8	2.2	n.a.	7.2	6.8	8.4	27.3
Anti-DP	13.6	41.6	7.6	n.a.	20.0	23.2	30.8	77.8
ΣDP	18.2	56.4	9.8	n.a.	27.2	30.0	39.2	105.1
ΣHFR	72.3	235.6	99.9	n.a.	812.3	139.2	331.6	1152.9

Table S 19: Concentrations (ng/g dw) of PBBs in investigated moss samples

	BG	SY	L	BL	SL-SB	SL-WA	SO	HA
PBB 52	< 0.0209	< 0.0227	< 0.0208	< 0.0233	< 0.0229	< 0.0214	< 0.0216	< 0.153
PBB 101	< 0.0314	< 0.0339	< 0.0312	< 0.0349	< 0.0345	< 0.0322	< 0.0323	< 0.2295
PBB 153	< 0.0523	< 0.0566	< 0.052	< 0.0584	< 0.0574	< 0.0536	< 0.0538	< 0.3826
PBB 180	< 0.3139	< 0.3391	< 0.3118	< 0.3494	< 0.3448	< 0.3219	< 0.323	< 2.2953
PBB 194	< 0.7093	< 0.5656	< 0.5201	< 1.0393	< 0.8594	< 0.9474	< 0.5376	< 3.8255
PBB 206	< 1.0463	< 1.1328	< 1.0414	< 1.1629	< 1.1458	< 1.0702	< 1.073	< 7.651
PBB 209	< 2.0926	< 2.2656	< 2.0828	< 2.3315	< 2.2917	< 2.1404	< 2.1567	< 15.302

Table S 20: Concentrations (ng/g fw) of PBBs in investigated moss samples

	BG	SY	L	BL	SL-SB	SL-WA	SO	HA
PBB 52	< 0.00226	< 0.00290	< 0.00352	< 0.00415	< 0.00220	< 0.00244	< 0.00201	< 0.0228
PBB 101	< 0.00339	< 0.00434	< 0.00527	< 0.00622	< 0.00331	< 0.00367	< 0.00301	< 0.0342
PBB 153	< 0.00565	< 0.00724	< 0.00879	< 0.0104	< 0.00551	< 0.00611	< 0.00501	< 0.0570
PBB 180	< 0.0339	< 0.0434	< 0.0527	< 0.0622	< 0.0331	< 0.0367	< 0.0301	< 0.342
PBB 194	< 0.0766	< 0.0724	< 0.0879	< 0.185	< 0.0825	< 0.108	< 0.0501	< 0.570
PBB 206	< 0.113	< 0.145	< 0.176	< 0.207	< 0.110	< 0.122	< 0.100	< 1.14
PBB 209	< 0.226	< 0.290	< 0.352	< 0.415	< 0.220	< 0.244	< 0.201	< 2.28

Table S 21: Concentrations (ng/g dw) of PFASs in investigated moss samples

	BG	SY	L	BL	SL-SB	SL-WA	SO	HA
PFBS	< 0.1556	< 0.168	< 0.1574	< 0.1478	< 0.1646	< 0.1544	< 0.1513	< 0.1557
PFHxS	< 0.1556	< 0.168	< 0.1574	< 0.1478	< 0.1646	< 0.1544	< 0.1513	< 0.1557
PFHpS	< 0.1556	< 0.168	< 0.1574	< 0.1478	< 0.1646	< 0.1544	< 0.1513	< 0.1557
PFOS	< 0.1037	< 0.1125	< 0.1047	0.224	< 0.1094	< 0.1026	< 0.1009	< 0.104
PFDS	< 0.1556	< 0.168	< 0.1574	< 0.1478	< 0.1646	< 0.1544	< 0.1513	< 0.1557
PFBA	< 0.1037	< 0.1125	< 0.1047	< 0.0983	< 0.1094	< 0.1026	< 0.1009	< 0.104
PFPA	< 0.1037	< 0.1125	< 0.1047	< 0.0983	< 0.1094	< 0.1026	< 0.1009	< 0.104
PFHxA	< 0.1037	< 0.1125	< 0.1047	< 0.0983	< 0.1094	< 0.1026	< 0.1009	< 0.104
PFHpA	< 0.1037	< 0.1125	< 0.1047	< 0.0983	< 0.1094	< 0.1026	< 0.1009	< 0.104
PFOA	< 0.1037	< 0.1125	< 0.1047	< 0.0983	< 0.1094	< 0.1026	< 0.1009	< 0.104
PFNA	< 0.1037	< 0.1125	< 0.1047	< 0.0983	< 0.1094	< 0.1026	< 0.1009	< 0.104
PFDA	< 0.1037	< 0.1125	< 0.1047	< 0.0983	< 0.1094	< 0.1026	< 0.1009	< 0.104
PFUnA	< 0.1037	< 0.1125	< 0.1047	< 0.0983	< 0.1094	< 0.1026	< 0.1009	< 0.104
PFDOA	< 0.1037	< 0.1125	< 0.1047	< 0.0983	< 0.1094	< 0.1026	< 0.1009	< 0.104
PFTrA	< 0.1037	< 0.1125	< 0.1047	< 0.0983	< 0.1094	< 0.1026	< 0.1009	< 0.104
PFTeA	< 0.1037	< 0.1125	< 0.1047	< 0.0983	< 0.1094	< 0.1026	< 0.1009	< 0.104

Table S 22: Concentrations (ng/g dw) of PFASs in investigated moss samples

	BG	SY	L	BL	SL-SB	SL-WA	SO	HA
PFBS	< 0.0168	< 0.0215	< 0.0266	< 0.0263	< 0.0158	< 0.0176	< 0.0141	< 0.0232
PFHxS	< 0.0168	< 0.0215	< 0.0266	< 0.0263	< 0.0158	< 0.0176	< 0.0141	< 0.0232
PFHpS	< 0.0168	< 0.0215	< 0.0266	< 0.0263	< 0.0158	< 0.0176	< 0.0141	< 0.0232
PFOS	< 0.0112	< 0.0144	< 0.0177	0.040	< 0.0105	< 0.0117	< 0.00943	< 0.0155
PFDS	< 0.0168	< 0.0215	< 0.0266	< 0.0263	< 0.0158	< 0.0176	< 0.0141	< 0.0232
PFBA	< 0.0112	< 0.0144	< 0.0177	< 0.0175	< 0.0105	< 0.0117	< 0.00943	< 0.0155
PFPA	< 0.0112	< 0.0144	< 0.0177	< 0.0175	< 0.0105	< 0.0117	< 0.00943	< 0.0155
PFHxA	< 0.0112	< 0.0144	< 0.0177	< 0.0175	< 0.0105	< 0.0117	< 0.00943	< 0.0155
PFHpA	< 0.0112	< 0.0144	< 0.0177	< 0.0175	< 0.0105	< 0.0117	< 0.00943	< 0.0155
PFOA	< 0.0112	< 0.0144	< 0.0177	< 0.0175	< 0.0105	< 0.0117	< 0.00943	< 0.0155
PFNA	< 0.0112	< 0.0144	< 0.0177	< 0.0175	< 0.0105	< 0.0117	< 0.00943	< 0.0155
PFDA	< 0.0112	< 0.0144	< 0.0177	< 0.0175	< 0.0105	< 0.0117	< 0.00943	< 0.0155
PFUnA	< 0.0112	< 0.0144	< 0.0177	< 0.0175	< 0.0105	< 0.0117	< 0.00943	< 0.0155
PFDOA	< 0.0112	< 0.0144	< 0.0177	< 0.0175	< 0.0105	< 0.0117	< 0.00943	< 0.0155
PFTrA	< 0.0112	< 0.0144	< 0.0177	< 0.0175	< 0.0105	< 0.0117	< 0.00943	< 0.0155
PFTeA	< 0.0112	< 0.0144	< 0.0177	< 0.0175	< 0.0105	< 0.0117	< 0.00943	< 0.0155

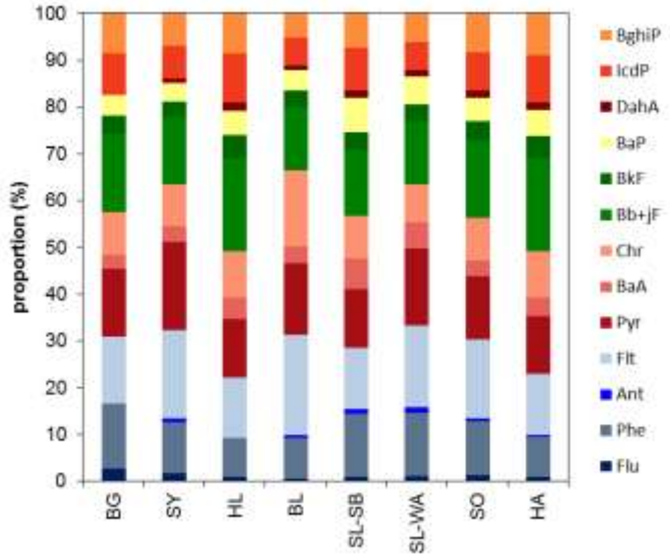

Figure S 1: Composition (%) of PAH quantified in investigated moss samples

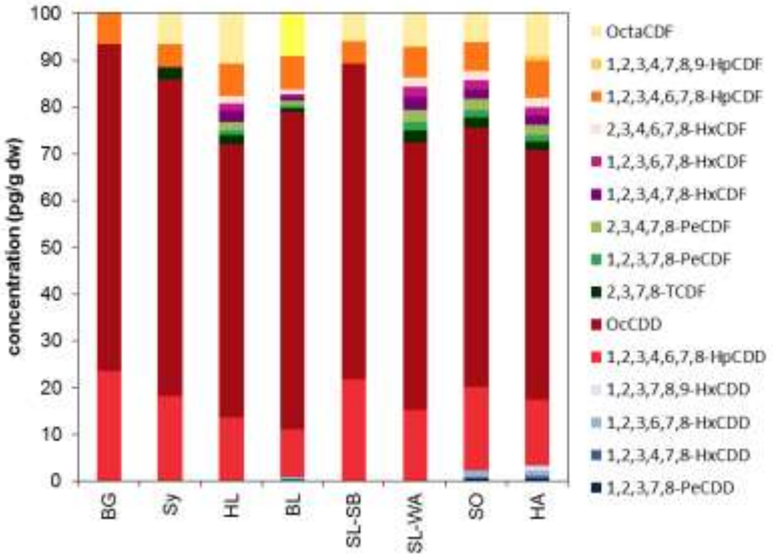

Figure S 2: Composition (%) of PCDD/F quantified in investigated moss samples

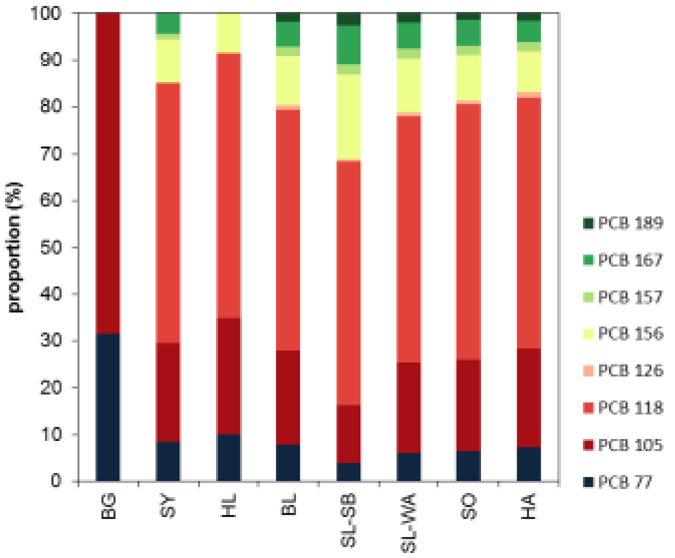

Figure S 3: Composition (%) of dl-PCB quantified in investigated moss samples

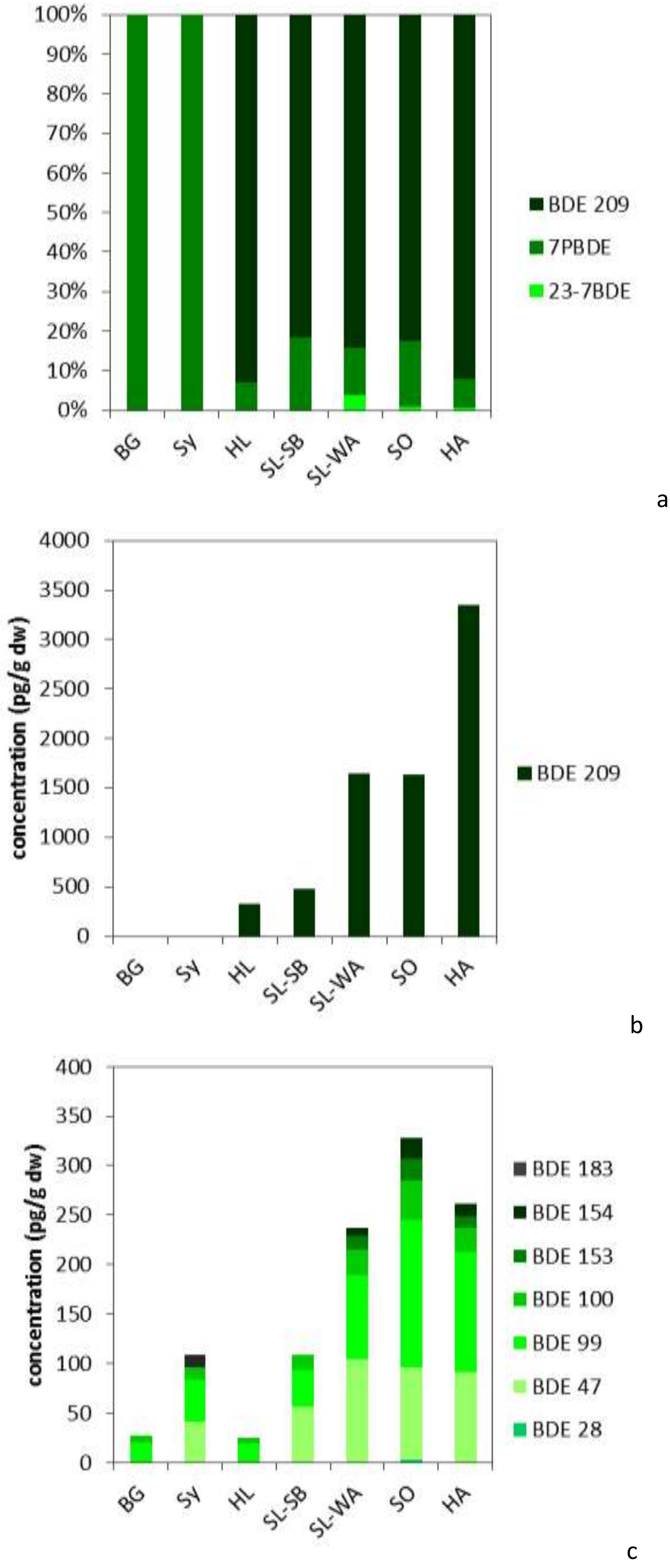

Figure S 4: Composition (%;a) and concentrations (pg/g dw; b, c) of PBDE in investigated moss samples

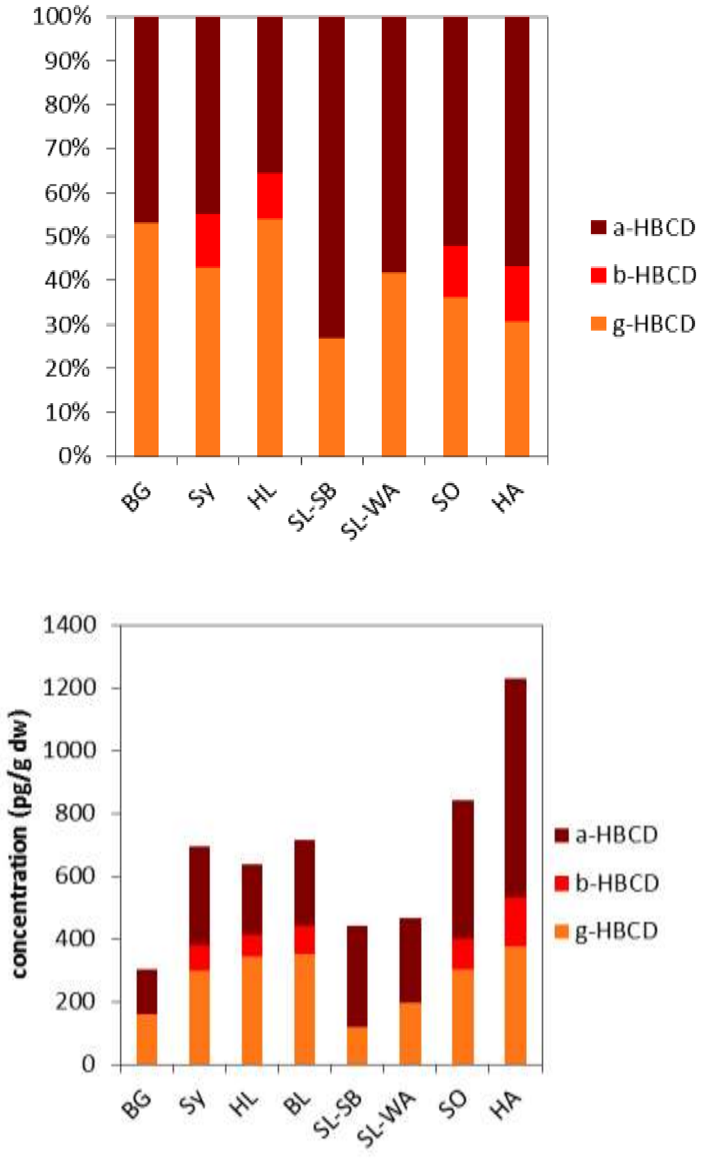

Figure S 5: Composition (%; a) and concentrations (pg/g dw; b) of HBCD in investigated moss samples

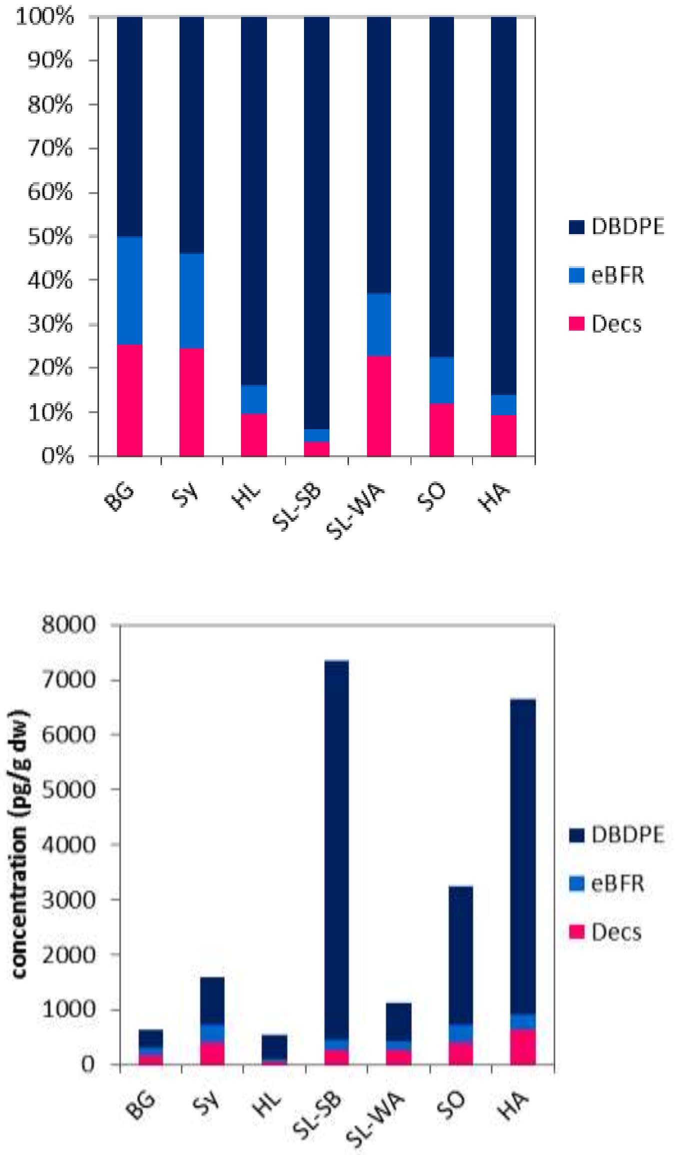

Figure S 6: Composition (%; a) and concentrations (pg/g dw; b) of HFR in investigated moss samples

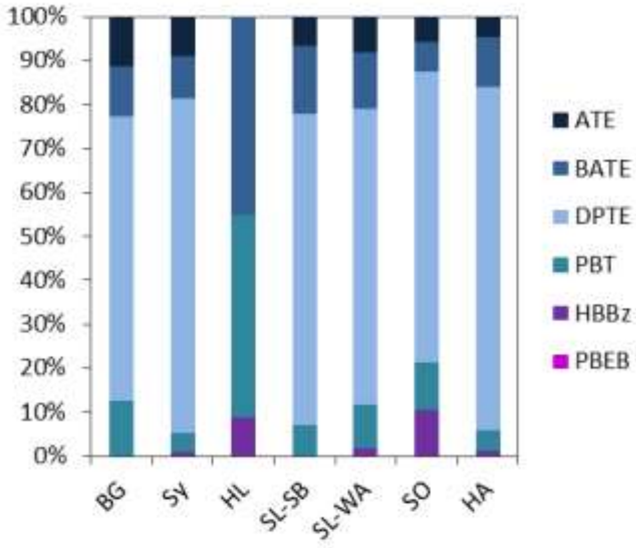

Figure S 7: Composition (%) of emerging brominated flame retardants in investigated moss samples

Table S 23: Diagnostic ratios of certain PAH

	BG	Sy	L	BL	SL-SB	SL-WA	SO	HA
Fla/(Fla+Pyr)	0.49	0.50	0.51	0.59	0.51	0.52	0.56	0.52
BaA/(BaA+Chr)	0.24	0.27	0.31	0.18	0.42	0.40	0.28	0.29
IcdP/IcdP+BghiP	0.51	0.50	0.56	0.54	0.55	0.49	0.50	0.53
BaP/BghiP	0.51	0.57	0.62	0.82	1.01	0.96	0.60	0.62

Flu/(Flu+Pyr)	*<0.5 petrol emi; >0.5 diesel emission*
Fla/(Fla+Pyr)	*<0.4 petrogenic; 0.4--0.5 fossil fuel comb; >0.5 grass/wood/coal comb.*
BaA/(BaA+Chr)	*<0.2 petrogenic; 0.2-0.35 coal comb; >0.35 vehicular emi. combustion*
IcdP/IcdP+BghiP	*<0.2 petrogenic; 0.2-0.5 petroleum combustion; >0.5 gras/wood/coal combustion*
BaP/BghiP	*<0.6 non traffic emi; > 0.6 traffic emi*

(Tobiszewski and Namiesnik, 2011)

Comparison of PAH in moss and tree leaf samples

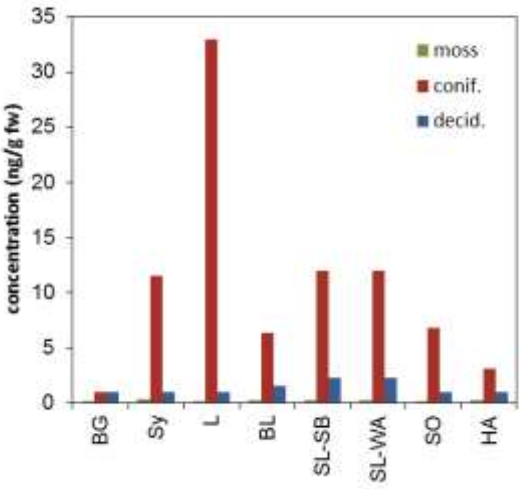

Phe

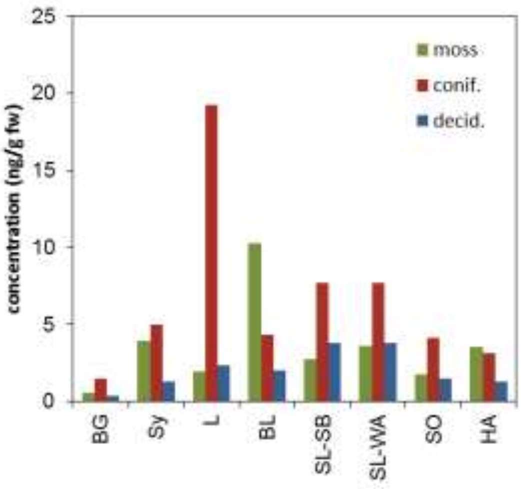

Fla

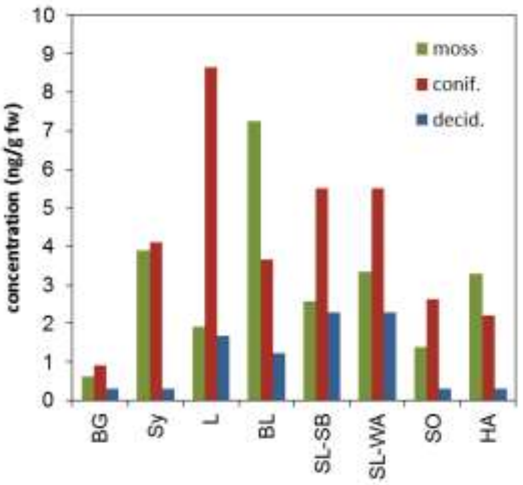

Pyr

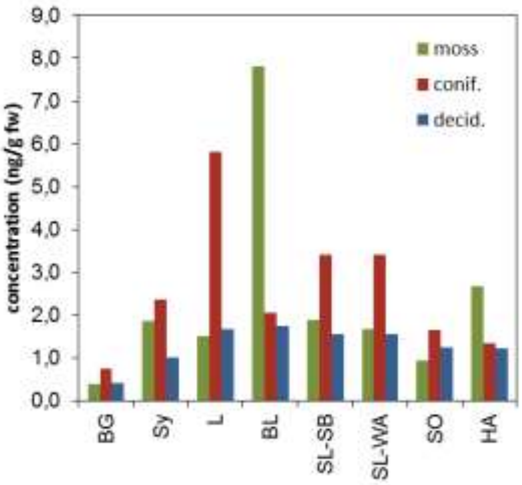

Chr

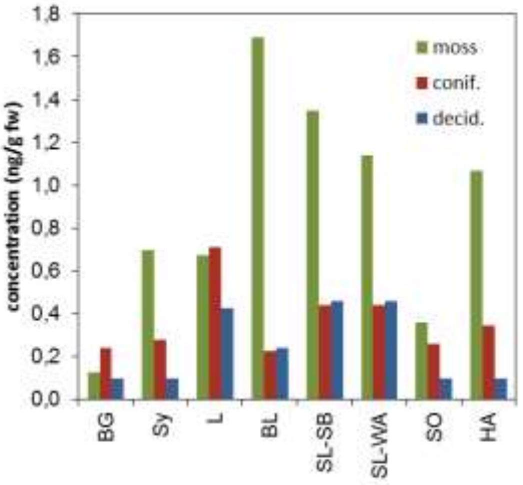

BaA

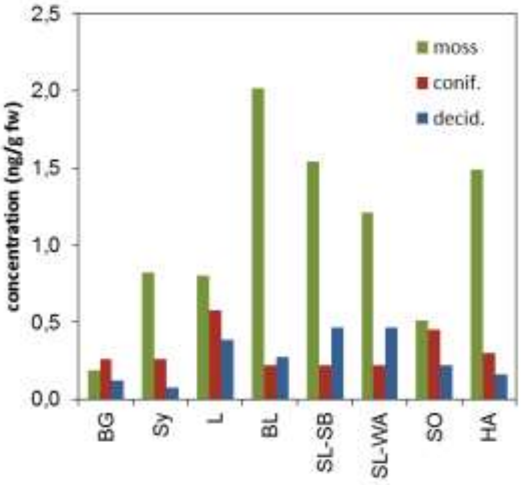

BaP

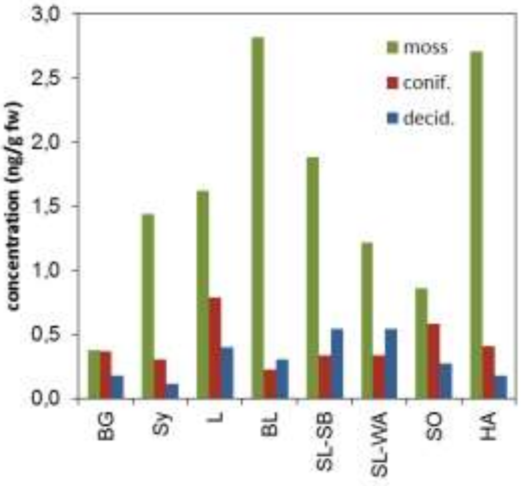

IcdP

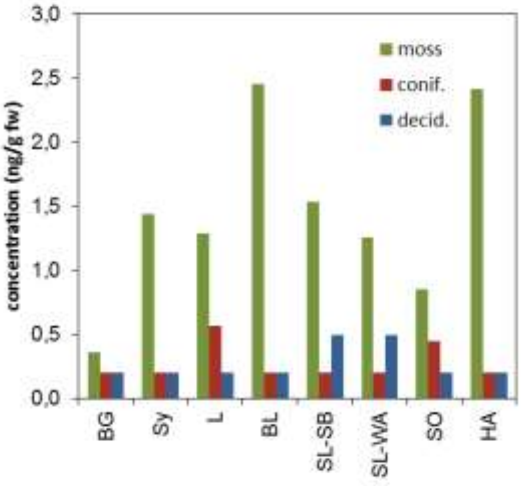

BghiP

Figure S 8: Comparison of PAH concentrations in moss, coniferous shoots and deciduous tree leaves

Comparison of flame retardants in moss and tree leaf samples

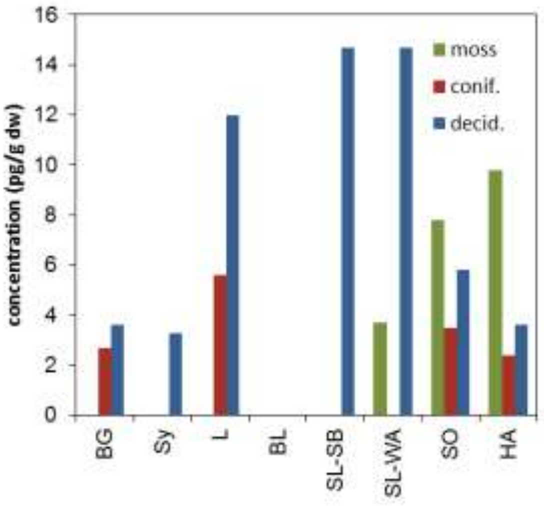

BDE 49

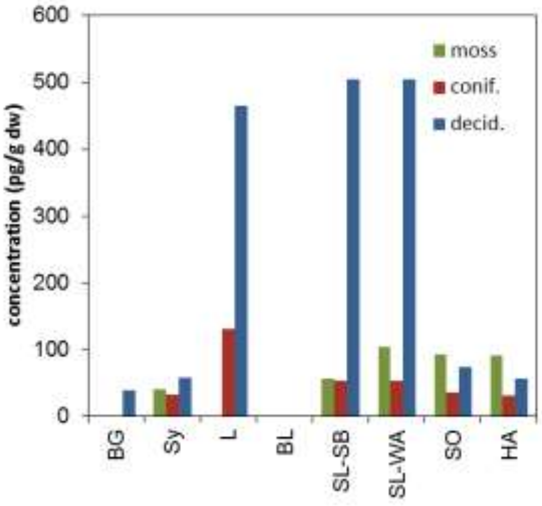

BDE47

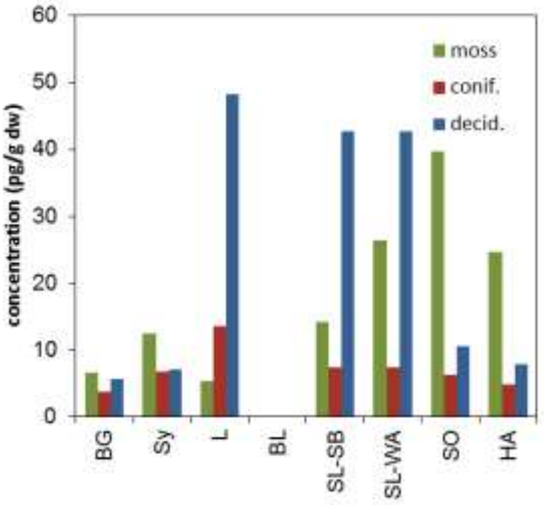

BDE100

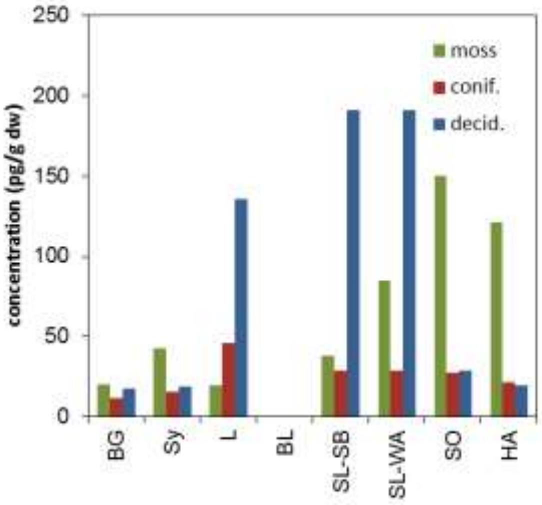

BDE99

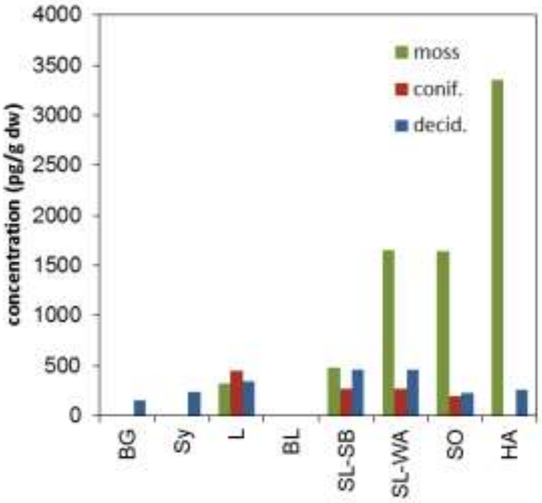

BDE 209

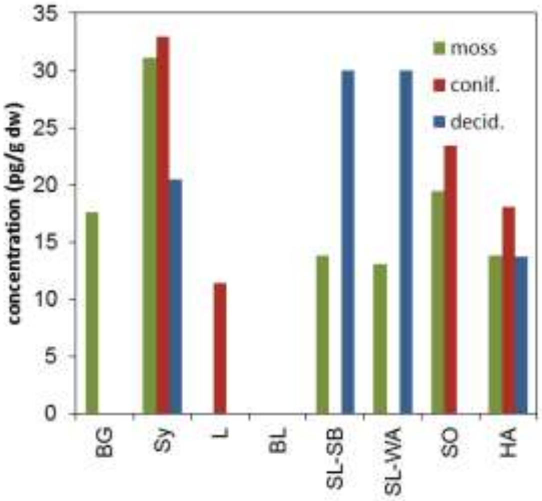

ATE

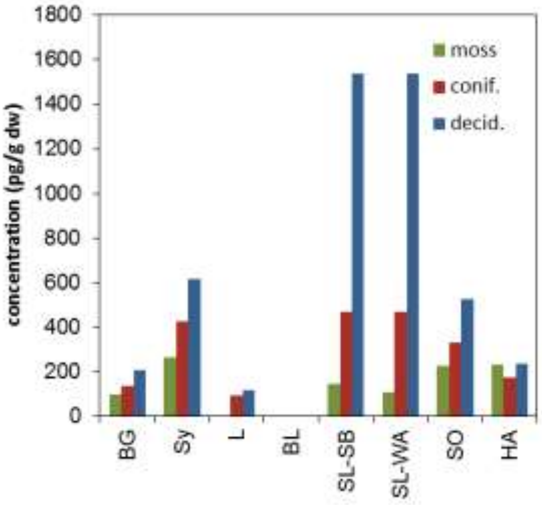

DPTE

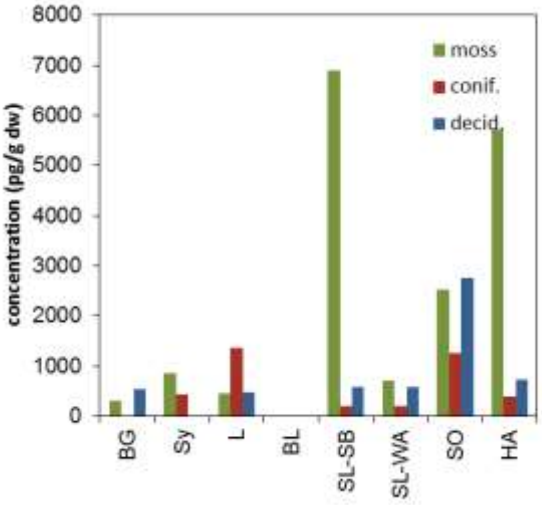

DBDPE

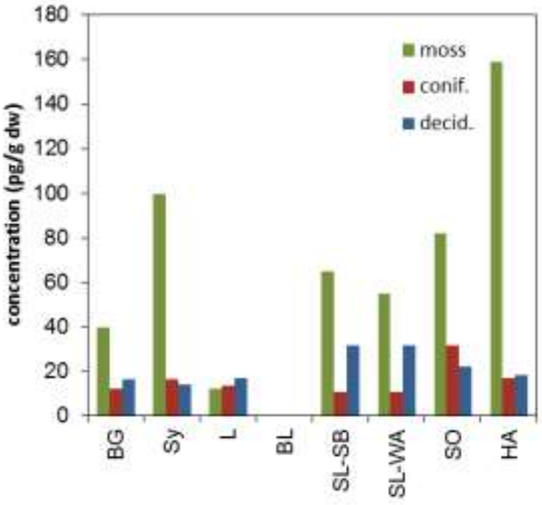

syn-DP

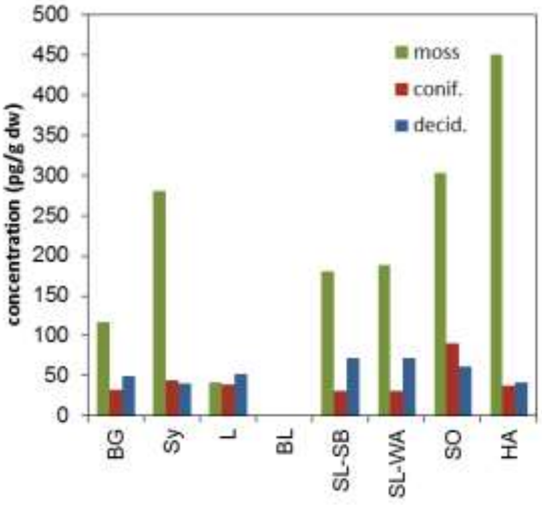

anti-DP

Figure S 9: Comparison of FR concentrations in moss, coniferous shoots and deciduous tree leaves

References

Tobiszewski, M. and Namiesnik, J., 2011. PAH diagnostic ratios for the identification of pollution emission sources. Environmental Pollution, 162: 110-119.
